# Crisaborole-Loaded Nanoemulsion for Atopic Dermatitis: Localized Dermal Delivery of a Phosphodiesterase-4 Inhibitor

**DOI:** 10.3390/pharmaceutics18101342

**Published:** 2026-10-21

**Authors:** Lupe Carolina Espinoza, Lilian Sosa, Paulo Sarango-Granda, Elena Sánchez-López, Jandry Rosales-Sánchez, Dagmar Gualotuña Campoverde, Rafaela Polo, Mireia Mallandrich, Ana Cristina Calpena

**Affiliations:** 1 Departamento de Química, Facultad de Ciencias Exactas y Naturales, Universidad Técnica Particular de Loja, Loja 110107, Ecuador; pcsarango@utpl.edu.ec (P.S.-G.); jxrosales@utpl.edu.ec (J.R.-S.); degualotuna1@utpl.edu.ec (D.G.C.); mrpolo@utpl.edu.ec (R.P.); 2 Universidad Pedagógica Nacional Francisco Morazán, Tegucigalpa 11101, Honduras; 3 Instituto de Investigaciones en Microbiología (IIM), Facultad de Ciencias, Universidad Nacional Autónoma de Honduras (UNAH), Tegucigalpa 11101, Honduras; 4 Facultad de Ciencias Químicas y Farmacia, Universidad Nacional Autónoma de Honduras (UNAH), Tegucigalpa 11101, Honduras; 5 Department of Pharmacy and Pharmaceutical Technology and Physical Chemistry, Faculty of Pharmacy and Food Sciences, University of Barcelona, 08028 Barcelona, Spain; esanchezlopez@ub.edu (E.S.-L.); mireia.mallandrich@ub.edu (M.M.); anacalpena@ub.edu (A.C.C.); 6 Institute of Nanoscience and Nanotechnology (IN2UB), University of Barcelona, 08028 Barcelona, Spain

**Keywords:** crisaborole, nanoemulsion, inflammation, skin, atopic dermatitis

## Abstract

**Background/Objectives**: Crisaborole (CRB) is a phosphodiesterase-4 inhibitor used for the topical treatment of mild-to-moderate atopic dermatitis (AD). However, its low aqueous solubility presents challenges for topical formulation and localized drug delivery. This study aimed to develop a CRB-loaded nanoemulsion (CRB-NE) and evaluate its physicochemical and biopharmaceutical characteristics, tolerability, and anti-inflammatory activity. **Methods**: CRB-NE (0.5% *w*/*w*) was developed using pseudo-ternary phase diagrams. Droplet size, morphology, rheological behavior, spreadability, and stability were characterized. Drug release and ex vivo permeation in human skin were evaluated. Biological compatibility was investigated using HaCaT keratinocytes and the hen’s egg test–chorioallantoic membrane (HET-CAM) assay, while skin barrier compatibility was assessed in healthy volunteers. Anti-inflammatory activity was evaluated through IL-8 secretion in stimulated HaCaT cells and a xylene-induced ear inflammation model in mice. **Results**: CRB-NE showed a droplet size of 105.3 ± 6.7 nm, a polydispersity index of 0.287 ± 0.012, Newtonian flow behavior, and physical stability over 60 days. Approximately 80% of CRB was released following first-order kinetics. After 24 h, ex vivo experiments showed cutaneous retention of 1098.43 μg CRB/g skin/cm^2^ and cumulative permeation of 23.75 ± 4.44 μg/cm^2^. Keratinocyte viability exceeded 80% at CRB concentrations of 2.5–10 μg/mL, and HET-CAM results indicated low irritation potential. The vehicle did not adversely affect skin barrier measurements in healthy volunteers. CRB-NE significantly reduced IL-8 secretion in TNF-α-stimulated keratinocytes and attenuated erythema, inflammatory cell infiltration, edema, vascular congestion, and tissue damage in vivo. **Conclusions**: CRB-NE demonstrated favorable physicochemical properties, substantial cutaneous drug retention, and anti-inflammatory activity, supporting its potential as a localized topical delivery system.

## 1. Introduction

Atopic dermatitis (AD) is a recurrent inflammatory disorder of the skin in which persistent itching, eczematous manifestations, and disruption of the epidermal barrier substantially affect patients’ quality of life. Although AD commonly develops during childhood, its manifestations may continue into adulthood or emerge later in life. The disease affects an estimated 15–20% of children and up to 10% of adults worldwide [1]. Its development cannot be attributed to a single mechanism; rather, it reflects the combined influence of genetic predisposition, altered epidermal integrity, dysregulated immune responses, changes in cutaneous microbial communities, and environmental triggers. These mechanisms contribute to a self-perpetuating cycle of barrier impairment and cutaneous inflammation that underlies the recurrent nature of the disease [2,3].

Management of AD therefore requires a multifaceted approach aimed at restoring skin barrier function, controlling inflammation and pruritus, and preventing disease exacerbations [4]. Daily application of moisturizers and emollients remains an essential component of care, whereas topical corticosteroids and calcineurin inhibitors are widely used to manage cutaneous inflammation [5,6]. Nevertheless, extended or inappropriate corticosteroid use may induce undesirable local effects, including skin atrophy, telangiectasia, and acneiform or rosacea-like eruptions, particularly in sensitive skin areas. Calcineurin inhibitors provide a steroid-sparing alternative but may cause transient burning or itching at the application site [6,7]. Patients with more extensive or refractory disease may require systemic treatments, including conventional immunosuppressants, biologic therapies, and Janus kinase (JAK) inhibitors [5]. Nevertheless, the chronic and relapsing nature of AD continues to create a need for effective and well-tolerated non-steroidal topical therapies suitable for local and potentially prolonged treatment [4,8].

Among the non-steroidal agents available for AD, crisaborole (CRB) acts through selective inhibition of phosphodiesterase-4 (PDE4), an enzyme responsible for the breakdown of intracellular cyclic adenosine monophosphate (cAMP). By reducing cAMP degradation, CRB influences inflammatory signaling and the expression of mediators involved in cutaneous immune responses [9]. Its topical administration allows pharmacological activity to be directed toward the skin while limiting systemic exposure. CRB has a molecular weight of 251.05 g/mol and a log P of approximately 2.8, physicochemical characteristics compatible with dermal delivery [10]. Clinical studies have demonstrated its efficacy and generally favorable safety profile in both pediatric and adult patients, supporting its use as a steroid-sparing topical therapeutic option for AD [11,12].

Despite these advantages, effective topical delivery of CRB remains dependent on its release from the formulation, partitioning into the stratum corneum, and subsequent distribution and retention within cutaneous tissues [13]. The stratum corneum represents the principal diffusional barrier to dermal drug delivery, while the low aqueous solubility of lipophilic drugs may further complicate their incorporation into topical formulations and availability at the site of action [14]. Accordingly, formulation strategies capable of maintaining CRB in a solubilized state, promoting its release and partitioning into the skin, and favoring local retention may be particularly advantageous for topical therapy. In this context, nanotechnology-based delivery systems offer opportunities to optimize the cutaneous delivery of poorly water-soluble drugs [15,16].

Several formulation and delivery strategies have been explored to improve dermal drug delivery, including conventional gels, transdermal patches, microneedles, and nanocarrier-based systems. Conventional gels are non-invasive, easy to apply, and generally well accepted by patients; however, their performance may be limited for poorly water-soluble drugs when the vehicle does not provide sufficient solubilization capacity. Transdermal patches can provide controlled and sustained drug release over prolonged periods, but they are primarily designed to promote drug transport across the skin and may therefore be less suited when preferential drug localization within cutaneous tissues is desired [13]. Microneedles can effectively overcome the stratum corneum barrier by creating transient microchannels, thereby facilitating drug penetration; however, they require specialized devices and involve physical disruption of the skin barrier [17]. In contrast, nanoemulsions provide a non-invasive approach with particular advantages for lipophilic and poorly water-soluble compounds, including high solubilization capacity, a large interfacial surface area, and intimate contact with the skin [18]. Moreover, appropriate selection of the oil phase, surfactant, and co-surfactant can facilitate drug release and partitioning into cutaneous tissues and potentially favor local drug retention. These characteristics are particularly relevant for CRB, given its limited aqueous solubility and the therapeutic objective of achieving local drug availability within the skin [19].

Nanoemulsions are finely dispersed systems in which one liquid phase is distributed as small droplets within another immiscible phase, with interfacial stabilization provided by surfactants and, in many formulations, co-surfactants. Their droplet dimensions are commonly within the nanometric range, often below 200 nm [20]. Surfactants facilitate dispersion by lowering oil–water interfacial tension and reducing the tendency of droplets to merge, whereas co-surfactants can further improve interfacial flexibility and facilitate the formation of nanosized droplets [21].

On this basis, the present study aimed to develop and characterize a CRB-loaded nanoemulsion (CRB-NE) designed to improve the dermal delivery and local drug availability, and to evaluate its physicochemical properties, skin retention, tolerability, and anti-inflammatory efficacy.

## 2. Materials and Methods

### 2.1. Materials

Crisaborol (CRB) or 5-(4-cyanophenoxy)-1,3-dihydro-1-hydroxy-[1,2]-benzoxaborole, C_14_H_10_BNO_3_ with a purity of 99.3%, was purchased from Shandong Octagon Chemicals Limited (Yantai, China). Propylene glycol monocaprylate type II (Capryol^®^ 90), polyglyceryl-3 dioleate (Plurol^®^ oleique CC497), diethylene glycol monoethyl ether (Transcutol^®^P) and propylene glycol monolaurate type II (Lauroglycol^®^90) were supplied by Gattefossé (Saint-Priest, France). Polyoxyethylene sorbitan monooleate (Tween^®^ 80), castor oil and oleic acid were purchased from Sigma-Aldrich (Darmstadt, Germany). Macrogol glycerol ricinoleate (Cremophor^®^ EL) was supplied by Fagron Iberica (Barcelona, Spain). Components for histological analysis were purchased from Sigma and Thermo Fisher Scientific (Barcelona, Spain). HPLC-grade water was obtained using the Barnstead™ MicroPure Water Purification Systems (Thermo Fisher Scientific, Waltham, MA, USA).

### 2.2. Validation of the Analytical Method

A high-performance liquid chromatography (HPLC) method was validated for the quantification of CRB. Analyses were performed using a Thermo Scientific Ultimate 3000 UHPLC system equipped with an Ultimate 3000 pump and a DAD 3000 photodiode array detector (Thermo Scientific, Waltham, MA, USA). Data acquisition and processing were carried out using Compass QuatAnalysis software version 2.2 (Thermo Scientific, Waltham, MA, USA). Chromatographic separation was achieved on a Kromasil C18 column (250 × 4.6 mm, 5 μm particle size). The mobile phase consisted of acetonitrile and water (70:30, *v*/*v*), previously filtered through a 0.45 μm PVDF membrane filter (Millipore Corp., Madrid, Spain). Isocratic elution was performed at a flow rate of 0.75 mL/min and a column temperature of 25 °C. The injection volume was 10 μL, and detection was carried out at 245 nm using UV absorbance.

The HPLC method was validated for linearity (1–100 μg/mL), accuracy, intra- and inter-day precision, robustness, specificity, and sensitivity in accordance with the Q2A and Q2B guidelines of the International Conference on Harmonization (ICH). Linearity was evaluated by least-squares regression and ANOVA. Precision and accuracy were expressed as relative standard deviation (RSD%) and relative error (RE%), respectively. Robustness was assessed by varying the mobile phase composition by ±1%, while specificity was evaluated by examining potential chromatographic interference from skin-derived components. The limits of detection (LOD) and quantification (LOQ) were calculated from the standard deviation of the calibration intercept and the slope, using factors of 3.3 and 10, respectively [22].

### 2.3. Solubility of the Drug

The solubility of CRB was evaluated in various oils (castor oil, Plurol^®^ Oleique CC497, Lauroglycol^®^ 90, oleic acid and Capryol^®^ 90), surfactants (Tween^®^ 80 and Cremophor^®^ EL), and co-surfactants (propylene glycol, polyethylene glycol, and Transcutol^®^ P). An excess amount of CRB was added to 2 g of each vehicle. The mixtures were magnetically stirred for 2 h at 25 °C and subsequently allowed to equilibrate for 24 h at room temperature. After equilibration, the samples were centrifuged for 20 min, and the resulting supernatants were diluted with methanol prior to analysis. The concentration of dissolved CRB was determined by UV–Vis spectrophotometry at 285 nm. Blank samples corresponding to each vehicle were prepared following the same procedure.

### 2.4. Pseudo-Ternary Phase Diagrams and Preparation of CRB-NE

Four pseudo-ternary phase diagrams were constructed by the aqueous titration method using the excipients that exhibited the highest solubilization capacity for CRB, namely Lauroglycol^®^ 90 as the oil phase, Tween^®^ 80 as the surfactant, and Transcutol^®^ P as the co-surfactant. The surfactant/co-surfactant mixture (S_mix_) was evaluated at different weight ratios (2:1, 1:1, 1:2, and 1:3, *w*/*w*). The nanoemulsion region of each phase diagram was determined by mixing the oil phase and S_mix_ at weight ratios ranging from 9:1 to 1:9 (*w*/*w*). Purified water was then added dropwise under continuous stirring until turbidity or phase separation was observed.

The pseudo-ternary phase diagram with the largest nanoemulsion-forming region was selected. Six candidate compositions were subsequently selected within this region, preferentially considering compositions with a higher aqueous-phase proportion and lower S_mix_ contents to minimize surfactant exposure and its potential contribution to topical irritation. CRB (5 mg/g) was then incorporated into each candidate formulation by dissolving the drug in the oil phase under magnetic stirring at 500 rpm for 15 min. Subsequently, the surfactant and co-surfactant were added, and stirring was continued for an additional 30 min until complete dissolution of CRB was achieved. Finally, purified water was gradually incorporated while maintaining constant magnetic stirring for 10 min, resulting in the formation of a homogeneous and translucent nanoemulsion. The six CRB-loaded formulations were maintained under observation for two weeks and evaluated for changes in pH, macroscopic phase separation, and visible drug precipitation.

### 2.5. Physicochemical Characterization

#### 2.5.1. pH Values, Droplet Size, and Polydispersity

The pH value was measured using a calibrated GLP 22 digital pH meter (Crison Instruments; Barcelona, Spain).

The droplet size and polydispersity index (PDI) were determined using undiluted samples at 25 °C by dynamic light scattering (DLS) using a Zetasizer Nano ZS (Malvern Instruments; Malvern, UK). Values were reported as mean ± standard deviation (SD) from three replicates (*n* = 3).

#### 2.5.2. Morphological Characterization

Morphological characterization of the undiluted CRB-NE was carried out by transmission electron microscopy (TEM) using a JEOL JEM-1010 electron microscope (JEOL Ltd., Tokyo, Japan). For sample preparation, the formulation was negatively stained with UranyLess^®^ and dried at room temperature for 24 h before imaging.

#### 2.5.3. Viscosity and Rheological Behavior

The rheological behavior of CRB-NE was evaluated 24 h after preparation using a Thermo Scientific Haake Rheostress 1 rheometer (Thermo Fisher Scientific, Karlsruhe, Germany) equipped with a parallel-plate geometry (Haake PPti60, 60 mm diameter) and a gap of 0.5 mm. Temperature was maintained at 25 °C using a Thermo Haake Phoenix II temperature-control unit coupled to a Haake C25P circulator. Data acquisition and analysis were performed using Haake RheoWin^®^ Job Manager v.4.0 and RheoWin^®^ Data Manager v.4.0 software. Viscosity and flow behavior were determined under rotational mode at 25 °C. A three-step shear rate program was applied: an increase from 0 to 100 s^−1^ over 3 min, maintenance at 100 s^−1^ for 1 min, and a decrease to 0 s^−1^ over 3 min. The experimental flow data were fitted to different rheological models to characterize the flow behavior of the formulations. Model fitting was assessed based on the correlation coefficient (r) and chi-square (χ^2^) values, and the model providing the best statistical fit was selected.

#### 2.5.4. Spreadability Assessment

The spreadability of CRB-NE was determined in triplicate using a parallel-plate method. Briefly, 0.10 g of the formulation was placed at the center of a glass plate, and a plastic sheet weighing 3 g was carefully positioned on top of the sample. After an equilibration period of 1 min, the diameter of the spread formulation was measured. Subsequently, additional weights (4, 5, 8, 13, 23, 50, 75 and g) were sequentially applied at 30 s intervals, and the spreading area was recorded after each load. Spreadability was expressed as the area covered by the formulation as a function of the applied weight and was calculated according to Equation (1):

*S* = *π* × *r*^2^ (1)

where S is the spreading area and r is the radius of the spread formulation.

### 2.6. Stability Evaluation

CRB-NE was subjected to both physical and chemical stability evaluations. Physical stability was monitored by multiple light scattering (MLS) analysis using a Turbiscan Lab^®^ Expert (Formulaction, L’Union, France) equipped with a pulsed near-infrared light source (λ = 880 nm). Samples (20 mL) were stored at 4, 25, and 40 °C, and transmission (T) profiles were recorded after 1 and 60 days of storage. Changes in T profiles were used to detect potential destabilization phenomena during storage. Chemical stability was evaluated by determining the CRB content by HPLC method and pH in nanoemulsion samples stored at 25 and 40 °C over a period of three months.

### 2.7. In Vitro Drug Release

The in vitro release of CRB from the nanoemulsion was evaluated using vertical Franz diffusion cells (FDC 400, Crown Glass, Somerville, NJ, USA) with a receptor volume of 14 mL and an effective diffusion area of 1 cm^2^. The receptor compartment was filled with an acetonitrile–water mixture (70:30, *v*/*v*) and maintained under sink conditions at 32 °C with continuous magnetic stirring at 500 rpm. A dialysis membrane (MWCO 12 kDa), previously hydrated in a methanol–water solution (1:1, *v*/*v*) for 24 h, was positioned between the donor and receptor compartments. Subsequently, 300 μL of CRB-NE was placed in the donor chamber. At predetermined time intervals, 300 μL aliquots were withdrawn from the receptor compartment and immediately replaced with an equal volume of fresh receptor medium to maintain a constant volume. The collected samples were analyzed by HPLC, and the results were expressed as mean ± standard deviation (SD) of three independent experiments. The cumulative amount of CRB released was plotted as a function of time, and the release profiles were fitted to different kinetic models, including first-order, Higuchi, exponential growth, hyperbolic, Weibull, and Korsmeyer–Peppas models. The most suitable model was selected according to the coefficient of determination (R^2^) and the Akaike Information Criterion (AIC).

### 2.8. Ex Vivo Skin Permeation

Human skin was obtained from a healthy 38-year-old female donor undergoing abdominoplasty at Hospital de Barcelona (SCIAS, Barcelona, Spain). Tissue collection was performed after obtaining written informed consent and approval from the Hospital de Barcelona Ethics Committee (Protocol No. 002, approved on 17 January 2020). Prior to the experiments, skin integrity was verified by measuring transepidermal water loss (TEWL) using a Tewameter^®^ TM 300 (Courage & Khazaka Electronics GmbH, Cologne, Germany). Only skin samples exhibiting TEWL values below 10 g/m^2^·h were considered suitable for the study. Dermatomed skin membranes (0.4 mm thickness) were mounted on Franz diffusion cells (14 mL receptor volume, 1 cm^2^ diffusion area). The receptor compartment was filled with a Transcutol^®^ P–water mixture (60:40, *v*/*v*) and maintained at 32 °C under continuous stirring at 500 rpm to ensure sink conditions. Subsequently, 300 μL of CRB-NE was applied to the donor compartment. At predetermined time points over a 24 h period, aliquots of 300 μL were withdrawn from the receptor phase and immediately replaced with an equal volume of fresh receptor medium. The collected samples were analyzed by HPLC to determine the amount of CRB permeated through the skin. After completion of the permeation experiment, the skin membranes were carefully removed from Franz diffusion cells, rinsed with distilled water to eliminate residual formulation, and cut according to the exposed diffusion area. The tissue samples were weighed, and the amount of CRB retained in the skin (*Q*_ret_) was extracted using 2 mL of methanol by sonication for 20 min. The resulting extracts were filtered and analyzed by HPLC. Results were expressed as mean ± standard deviation (SD) of six independent experiments. *Q*_ret_ (μg CRB/g skin/cm^2^) was normalized to both the mass of the analyzed skin sample and the exposed diffusion area and calculated according to Equation (2):

(2) $$Q_{\mathrm{ret}}=\;\frac{\mathrm{Amount}\;\mathrm{of}\;\mathrm{CRB}\;\mathrm{retained}}{\left(\mathrm{grams}\;\mathrm{of}\;\mathrm{skin}\times \;\mathrm{diffusion}\;\mathrm{area}\right)}$$

The cumulative amount of CRB permeated per unit area (μg/cm^2^) was plotted as a function of time. The steady-state flux (*J*_ss_, µg/cm^2^/h) was calculated from the slope of the linear portion of the cumulative permeation profile. The permeability coefficient (*K*_p_, cm/h) was calculated according to Equation (3):

(3) $$K_{p}=\frac{J_{\mathrm{ss}}}{C_{0}}$$

where *J*_ss_ is the steady-state flux and *C*_0_ is the initial CRB concentration.

### 2.9. Safety Evaluation

#### 2.9.1. In Vitro Cytotoxicity Assessment by MTT Assay

The cytotoxicity of CRB-NE was evaluated using the immortalized human keratinocyte cell line HaCaT (Cytion, Germany; catalog no. 300493; Cellosaurus CVCL_0038) cultured in DMEM GlutaMAX™ medium supplemented with fetal bovine serum (FBS), non-essential amino acids, penicillin (100 U/mL), streptomycin (100 μg/mL), and HEPES at 37 °C and 5% CO_2_. Cells were seeded in 96-well plates (2 × 10^5^ cells/mL; 100 μL/well) and incubated for 24 h until reaching 80–90% confluence. Subsequently, cells were exposed to CRB-NE (2.5–50 μg/mL) or Blank-NE for 24 h. After washing with phosphate-buffered saline (PBS), cells were incubated with MTT (5 mg/mL) for 3 h at 37 °C. Formazan crystals were dissolved in 100 μL of dimethyl sulfoxide (DMSO), and absorbance was measured at 560 nm using a Varioskan™ LUX microplate reader (Thermo Scientific, Waltham, MA, USA). Cell viability was calculated relative to untreated controls (100% viability) using Equation (4). All experiments were performed in triplicate.

(4) $$\mathrm{Cell}\;\mathrm{viability}=\left[\frac{\mathrm{ABS}\;\mathrm{treated}\;\mathrm{cells}}{\mathrm{ABS}\;\mathrm{control}\;\mathrm{cells}}\right]\times 100$$

#### 2.9.2. In Vitro Hen’s Egg Test on the Chorioallantoic Membrane (HET-CAM) Assay

The irritation potential of CRB-NE was assessed using the Hen’s Egg Test on the Chorioallantoic Membrane (HET-CAM). Fertilized chicken eggs were carefully opened to expose the chorioallantoic membrane (CAM) which was gently moistened with 0.9% (*w*/*v*) sodium chloride solution. Subsequently, 0.1 mL of CRB-NE was applied directly onto the CAM surface. The membrane was observed immediately for 5 min to monitor the occurrence of vascular responses, including hemorrhage, lysis, and coagulation. A 0.1 N sodium hydroxide solution and 0.9% sodium chloride solution were used as positive and negative controls, respectively. The irritation potential was expressed as the irritation score (IS), calculated according to Equation (5). Results were reported as mean ± SD and classified according to the categories shown in Table 1.

(5) $$\mathrm{IS}=\left[\frac{\left(301-\mathrm{hemorrhage}\;\mathrm{time}\right)}{300}\times 5\right]+\left[\frac{\left(301-\mathrm{lysis}\;\mathrm{time}\right)}{300}\times 7\right]+\left[\frac{\left(301-\mathrm{coagulation}\;\mathrm{time}\right)}{300}\times 9\right]$$

#### 2.9.3. Skin Tolerance and Barrier Function Assessment

The effects of Blank-NE on skin barrier function were assessed by measuring transepidermal water loss (TEWL) and stratum corneum hydration (SCH) in 10 healthy female volunteers aged 25–35 years. The study protocol was approved by the Ethics Committee of the University of Barcelona (IRB00003099, 30 January 2019) and conducted in accordance with the principles of the Declaration of Helsinki. Written informed consent was obtained from all participants before enrollment. Participants were instructed to avoid the application of creams, lotions, sunscreens, or other cosmetic products on the test area for at least 48 h prior to the study. Before measurements, volunteers were acclimatized for approximately 30 min in a controlled environment maintained at 25 °C and 55% relative humidity. TEWL and SCH values were recorded before formulation application (baseline) and at 0.5, 1.5, 2.5, 4, and 8 h following topical application of Blank-NE. TEWL measurements were performed using a Tewameter^®^ TM 300, while SCH was determined with a Corneometer^®^ CM 825 (Courage + Khazaka Electronic GmbH, Cologne, Germany).

### 2.10. Anti-Inflammatory Efficacy

#### 2.10.1. In Vitro Anti-Inflammatory Efficacy

The anti-inflammatory activity of CRB-NE was evaluated using HaCaT cells. Briefly, cells were seeded in 24-well plates at a density of 2 × 10^5^ cells/mL and cultured until reaching 80–90% confluence. Inflammation was induced by stimulation with TNF-α (50 ng/mL), and cells were subsequently treated with different concentrations of CRB-NE. Stimulated cells with TNF-α were used as the positive control, whereas untreated cells served as the negative control. After 24 h of incubation, culture supernatants were collected, centrifuged at 10,000× *g* for 15 min at 4 °C, and stored at −80 °C until analysis. The concentration of the pro-inflammatory cytokine interleukin-8 (IL-8) in the supernatants was quantified using commercially available enzyme-linked immunosorbent assay (ELISA) kits (BD Biosciences, San Jose, CA, USA) according to the manufacturer’s instructions. Cytokine levels were expressed as pg/mL, and the results are presented as mean ± SD of three independent experiments.

#### 2.10.2. In Vivo Anti-Inflammatory Activity

##### Study Design and Animals

The anti-inflammatory activity of CRB-NE was evaluated using a xylene-induced ear edema model in 10-week-old male mice (Mus musculus). All procedures were approved by the Animal Ethics Committee of the National Autonomous University of Honduras (CICUAL 004-2025; 2 June 2025). Animals were acclimatized for 7 days under a 12 h light/dark cycle with free access to food and water and randomly allocated into five groups (*n* = 6 per group): healthy untreated control, xylene-induced inflammation treated with PBS, CRB-NE, Blank-NE, or commercial diclofenac gel. Inflammation was induced by applying xylene to the left ear, followed by topical treatment after 25 min. Ear thickness was measured using a digital thickness gauge (Mitutoyo Corp., Kawasaki, Japan) before xylene application, immediately after inflammation induction, and 20 min after treatment. Edema was determined from the increase in ear thickness relative to baseline, and anti-inflammatory activity was assessed by the reduction in edema following treatment.

At the end of the experiment, animals were euthanized by cervical dislocation, and ear tissues were collected for histopathological analysis. Samples were fixed in 4% buffered formaldehyde for 24 h, paraffin-embedded, sectioned (5 μm), and stained with hematoxylin and eosin (H&E). Histological evaluation was performed using an Olympus BX41 microscope (Olympus Corp., Tokyo, Japan). Inflammatory infiltrate, edema, vascular congestion, and tissue alterations were independently assessed by two blinded evaluators using three images per sample and a four-point semi-quantitative scale (0 = absent, 1 = mild, 2 = moderate, and 3 = severe).

## 3. Results

### 3.1. Validation of Analytical Method

Validation of the analytical method was performed in order to obtain a reliable method for the quantification of CRB. The results showed compliance with ICH guidelines, exhibiting good linearity in the range of 1 to 100 μg/mL (r^2^ = 0.9999) in addition to compliance with precision, accuracy, robustness, sensitivity and specificity. Detailed results are shown in the Supplementary Materials.

### 3.2. Solubility of the Drug

**Figure 1:**
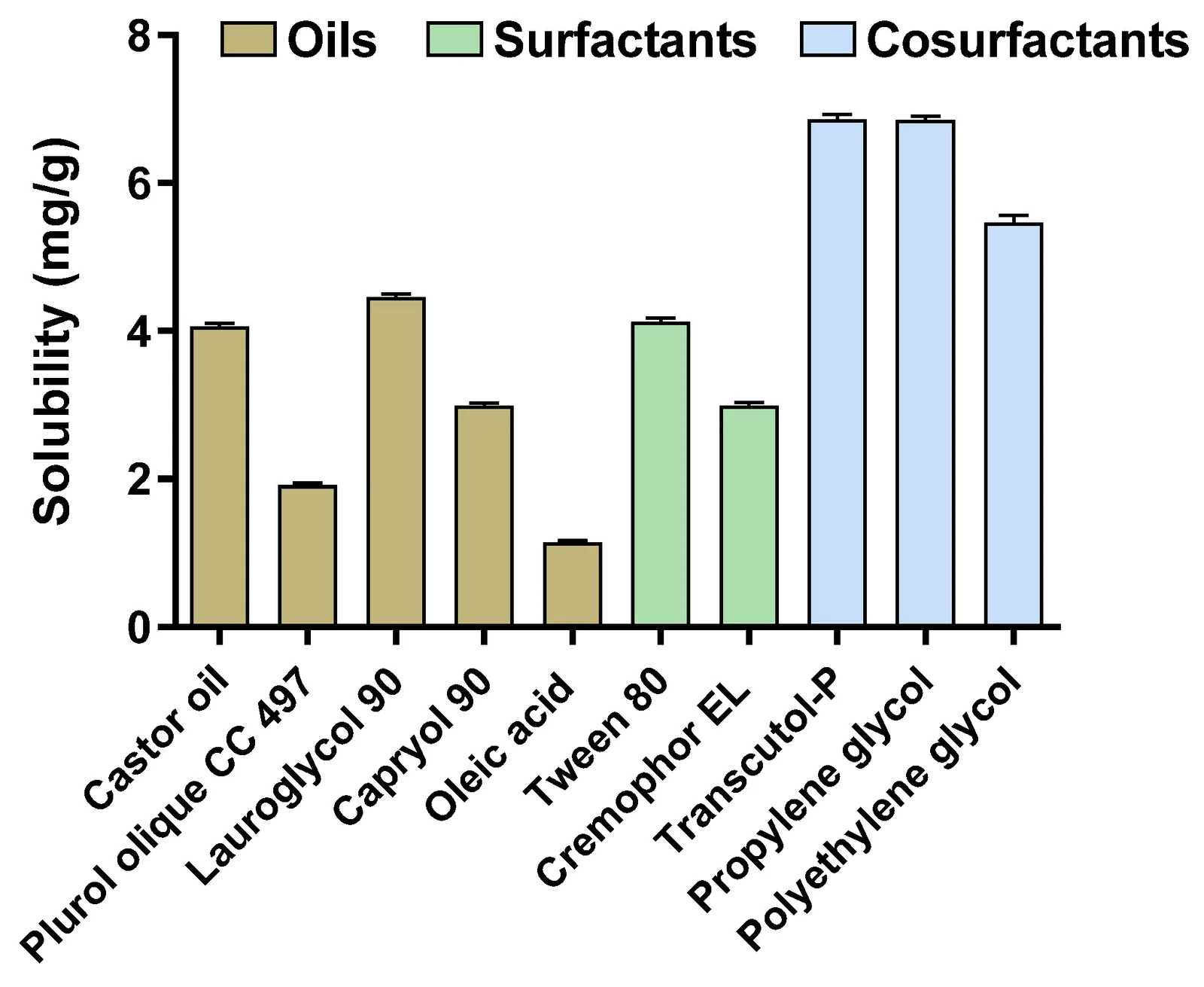
Solubility of crisaborole (CRB) in various oils, surfactants, and co-surfactants screened during excipient selection for nanoemulsion development. Results are presented as mean ± SD (*n* = 3).

The solubility of CRB in the different excipients evaluated is shown in Figure 1. Lauroglycol^®^ 90, Tween^®^ 80, and Transcutol^®^ P exhibited the highest solubilizing capacities among the oils, surfactants, and co-surfactants, with solubility values of 4.46, 4.13, and 6.86 mg/g, respectively. Based on these results, these excipients were selected for the subsequent development of the NE formulation.

### 3.3. Pseudo-Ternary Diagrams and Formulation of CRB-NE

**Figure 2:**
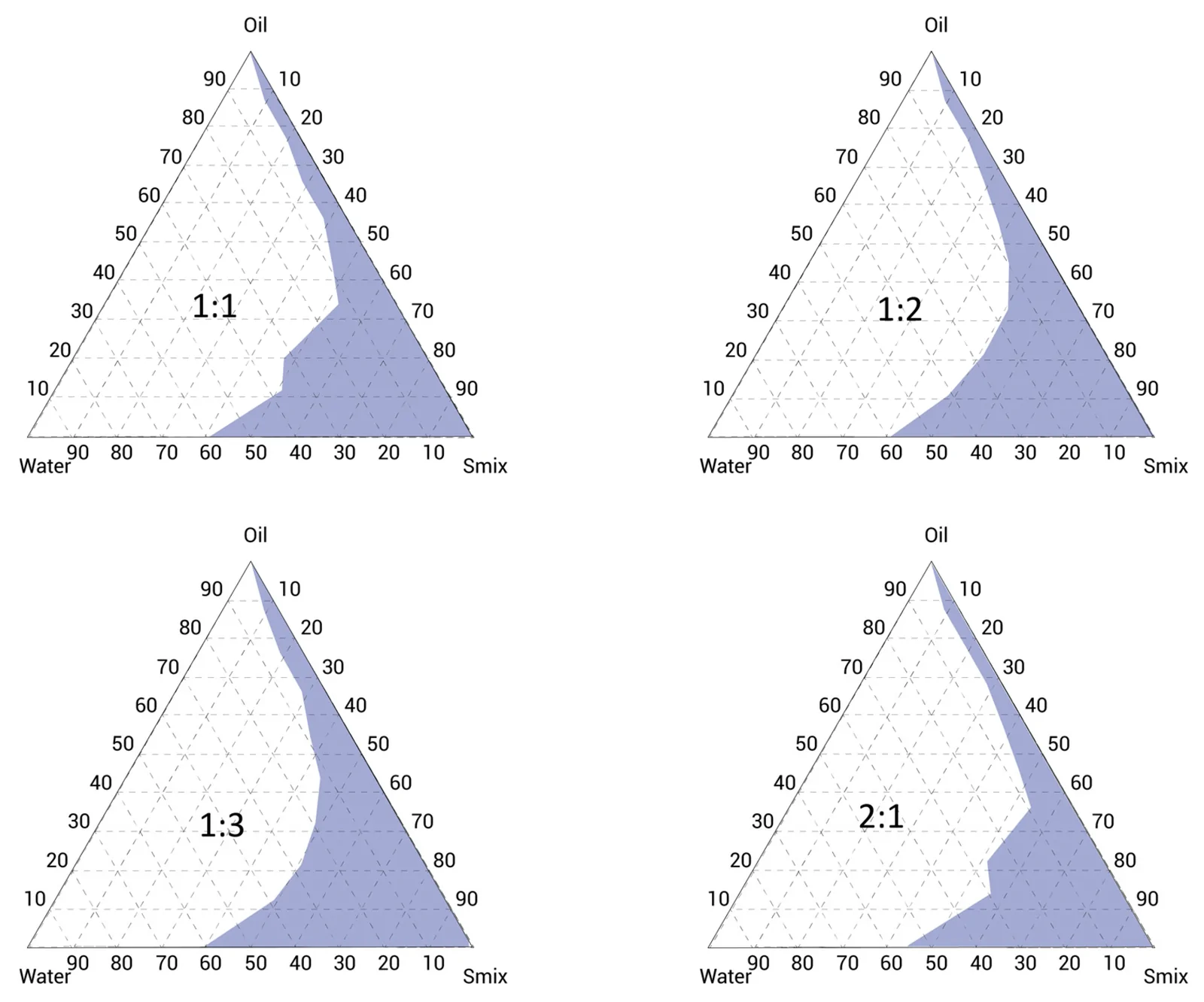
Pseudo-ternary phase diagrams constructed using Lauroglycol^®^ 90 as the oil phase and different Tween^®^ 80:Transcutol^®^ P ratios (S_mix_ 1:1, 1:2, 1:3, and 2:1). The shaded regions correspond to the compositions yielding transparent and homogeneous nanoemulsions.

Among the four pseudo-ternary phase diagrams evaluated, the S_mix_ composed of Tween^®^ 80 and Transcutol^®^ P at a 1:3 ratio exhibited the largest nanoemulsion region (Figure 2), indicating a greater capacity for spontaneous nanoemulsification. Consequently, this S_mix_ was selected for CRB incorporation.

The six candidate compositions are shown in the Supplementary Material (Table S5). As all candidate formulations remained physically stable throughout the observation period, the final selection was based on minimizing the S_mix_ content while maximizing the aqueous-phase proportion. Accordingly, F1 was selected as the optimized formulation.

Table 2 summarizes the composition of the final CRB-loaded nanoemulsion. The formulation consisted of 5 mg/g of CRB incorporated into a system containing 6% Lauroglycol^®^ 90 as the oil phase, 12.25% Tween^®^ 80 as the surfactant, 36.75% Transcutol^®^ P as the co-surfactant, and 45% purified water. The obtained system exhibited a translucent and homogeneous monophasic appearance, with no visible signs of phase separation or drug precipitation.

### 3.4. Physicochemical Characterization of CRB-NE

**Figure 3:**
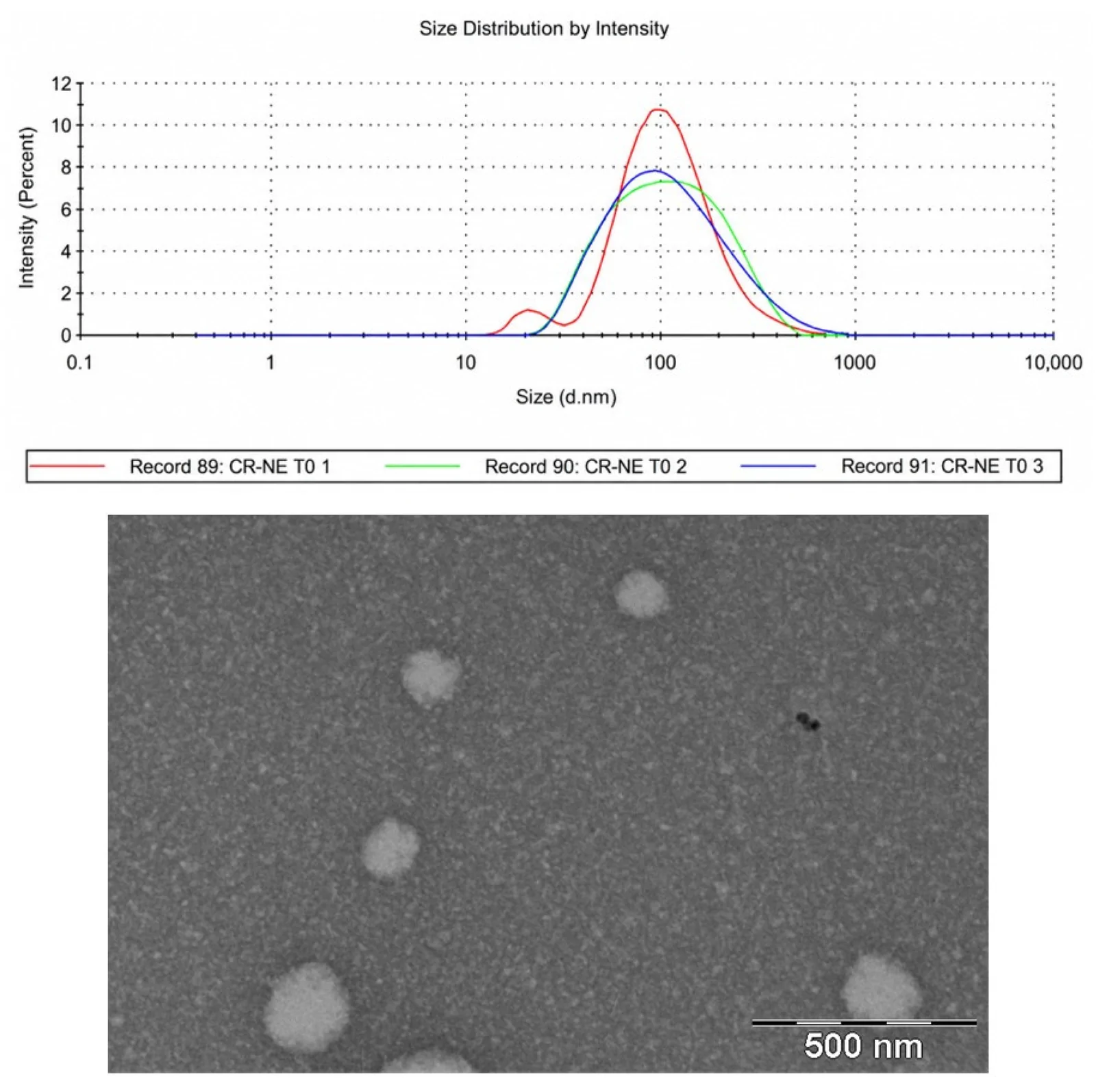
Droplet-size distribution determined by dynamic light scattering (DLS) and morphological examination of CRB-NE by Transmission electron microscopy (TEM).

CRB-NE exhibited a pH of 5.0 ± 0.02, which is compatible with the physiological pH of the skin. DLS analysis revealed a mean droplet size of 105.3 ± 6.7 nm and a PDI of 0.287 ± 0.012, indicating a narrow size distribution and good homogeneity of the NE. Morphological examination by TEM revealed spherical droplets, with dimensions in agreement with those obtained by DLS analysis (Figure 3).

**Figure 4:**
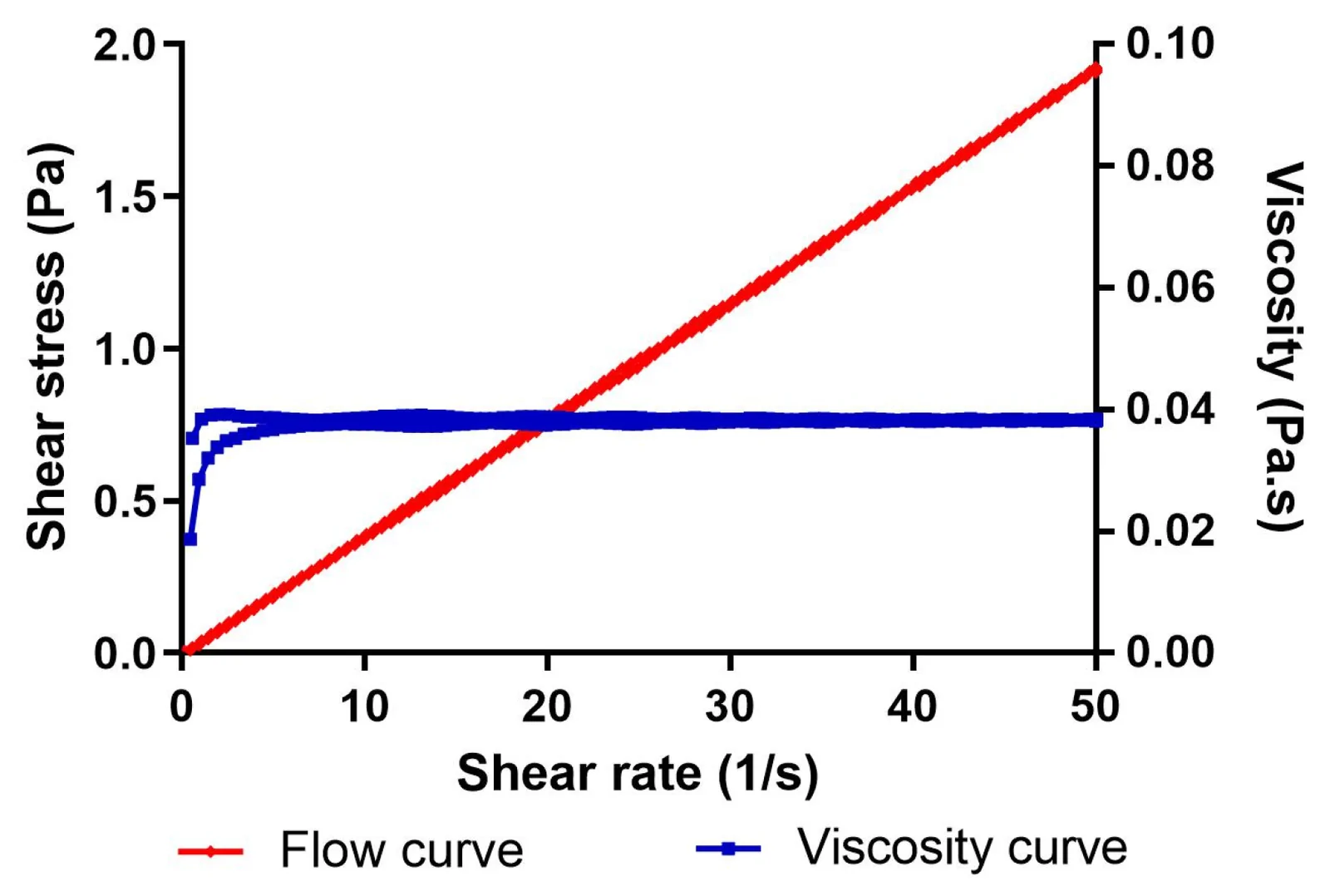
Rheological characterization of CRB-NE showing the flow curve (red) and viscosity profile (blue) as a function of shear rate.

The rheogram of CRB-NE (Figure 4) exhibited a linear relationship between shear stress and shear rate, while the viscosity remained essentially constant over the entire shear rate range evaluated. Regression analysis demonstrated that the experimental data were best fitted by the Newtonian model (R^2^ = 1). The mean viscosity of the formulation was 38.32 ± 0.04 mPa·s. In addition, no hysteresis loop was observed between the ascending and descending flow curves, indicating the absence of thixotropic behavior and confirming the Newtonian character of the system.

**Figure 5:**
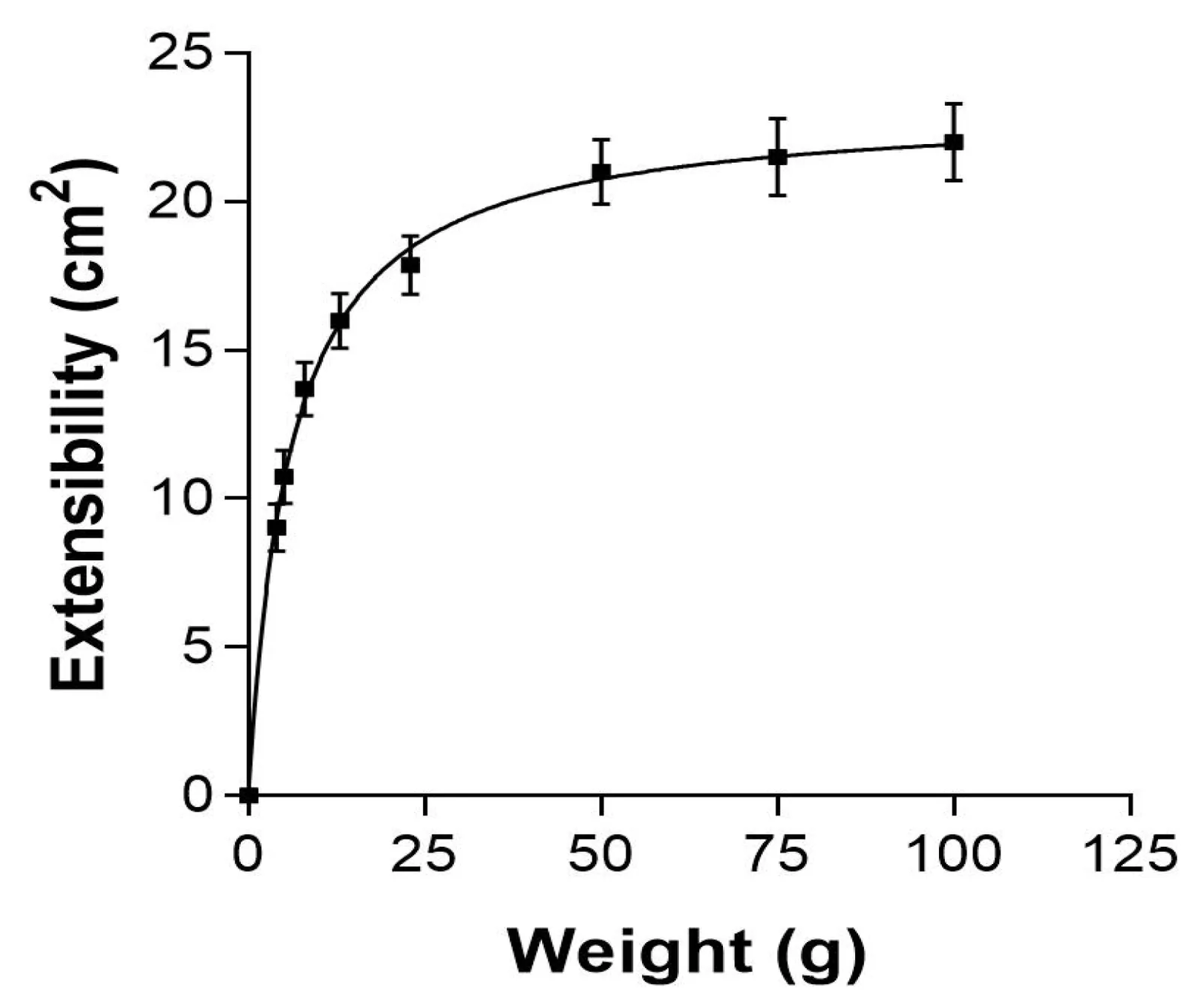
Spreadability profile of CRB-NE as a function of the applied weight. Data are expressed as mean ± SD (*n* = 3).

The spreadability study demonstrated that the spreading area of CRB-NE increased progressively with increasing applied weight (Figure 5). The formulation exhibited good spreading properties, reaching a maximum spreading area of approximately 22 cm^2^. Regression analysis indicated that the experimental data were best fitted by a hyperbolic model (R^2^ = 0.98).

### 3.5. Stability Studies

**Figure 6:**
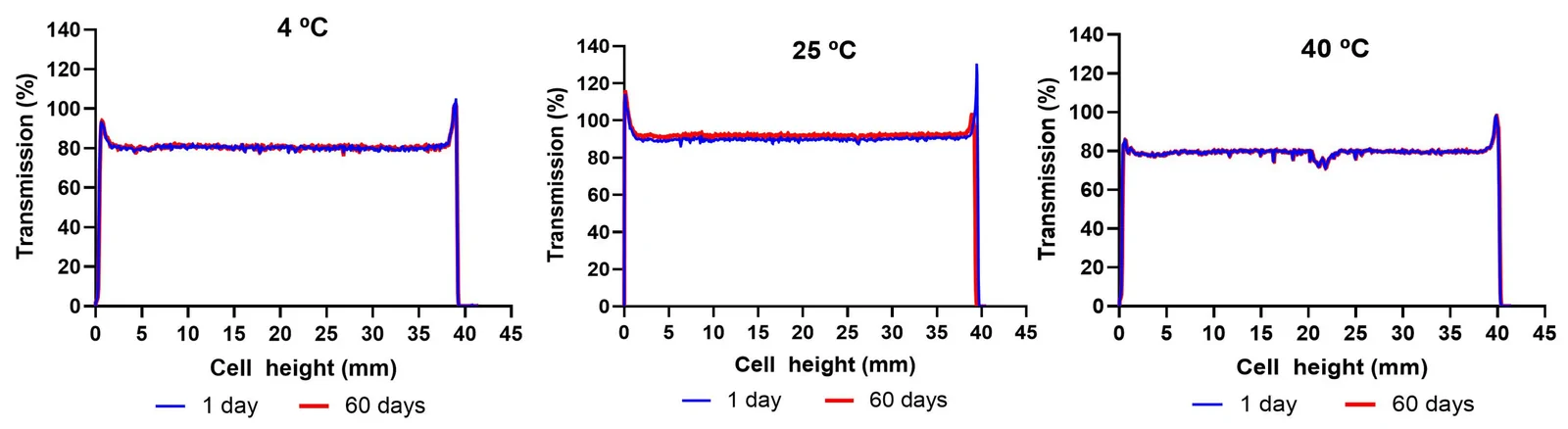
Multiple light scattering transmission profiles (%) obtained by Turbiscan^®^ analysis of CRB-NE after 60 days of storage at 4, 25, and 40 °C.

Figure 6 shows the transmission profiles (%) obtained for CRB-NE after 60 days of storage at 4, 25, and 40 °C. The peaks observed at both ends of the profiles correspond to the meniscus formed at the interface between the formulation and the sample vial. In Turbiscan^®^ analysis, variations in transmission signals greater than 10% are generally associated with destabilization phenomena. As shown in Figure 6, the transmission profiles remained unchanged throughout the storage period, indicating good physical stability of the formulation. No evidence of flocculation, coalescence, or precipitation was observed under any of the storage conditions evaluated.

**Figure 7:**
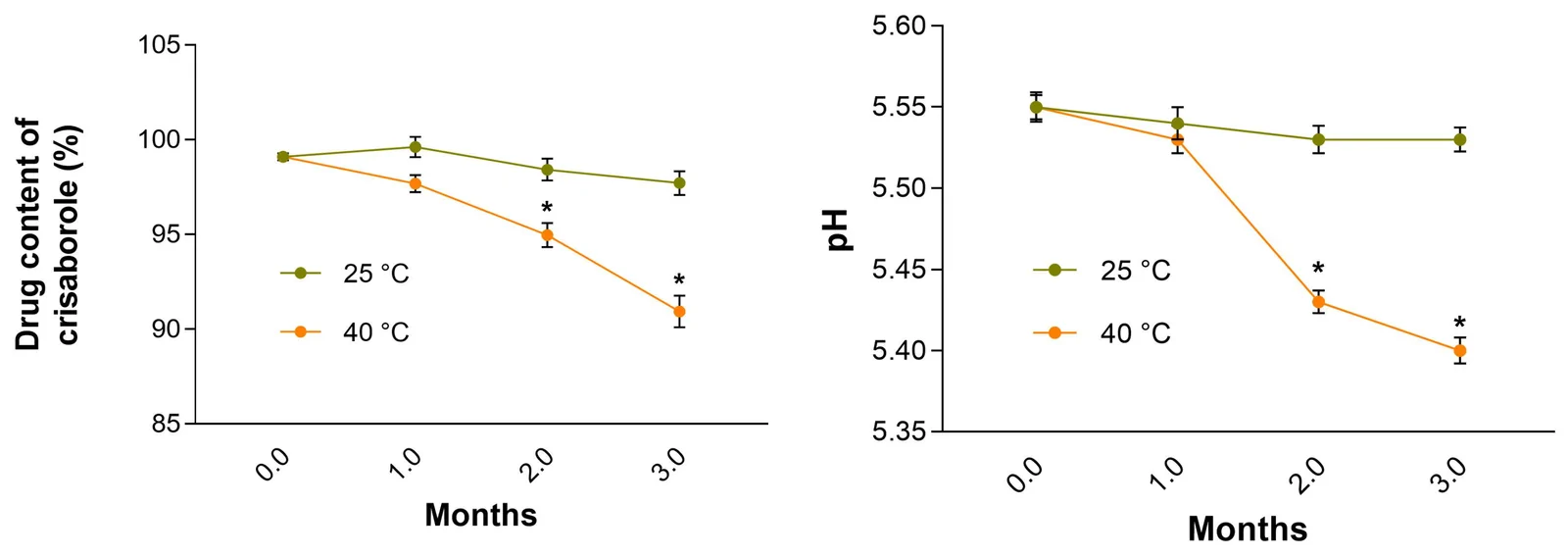
Evolution of pH and CRB content of CRB-NE during 3 months of storage at 25 and 40 °C. Values are expressed as mean ± SD (*n* = 3). Significant statistical differences * *p* < 0.05 comparison with the basal state.

As shown in Figure 7, CRB-NE maintained stable pH values and drug content during 3 months of storage period at 25 °C. However, exposure to 40 °C led to significant changes in both parameters, with noticeable alterations appearing after two months of storage.

### 3.6. In Vitro Release Study of CRB-NE

**Figure 8:**
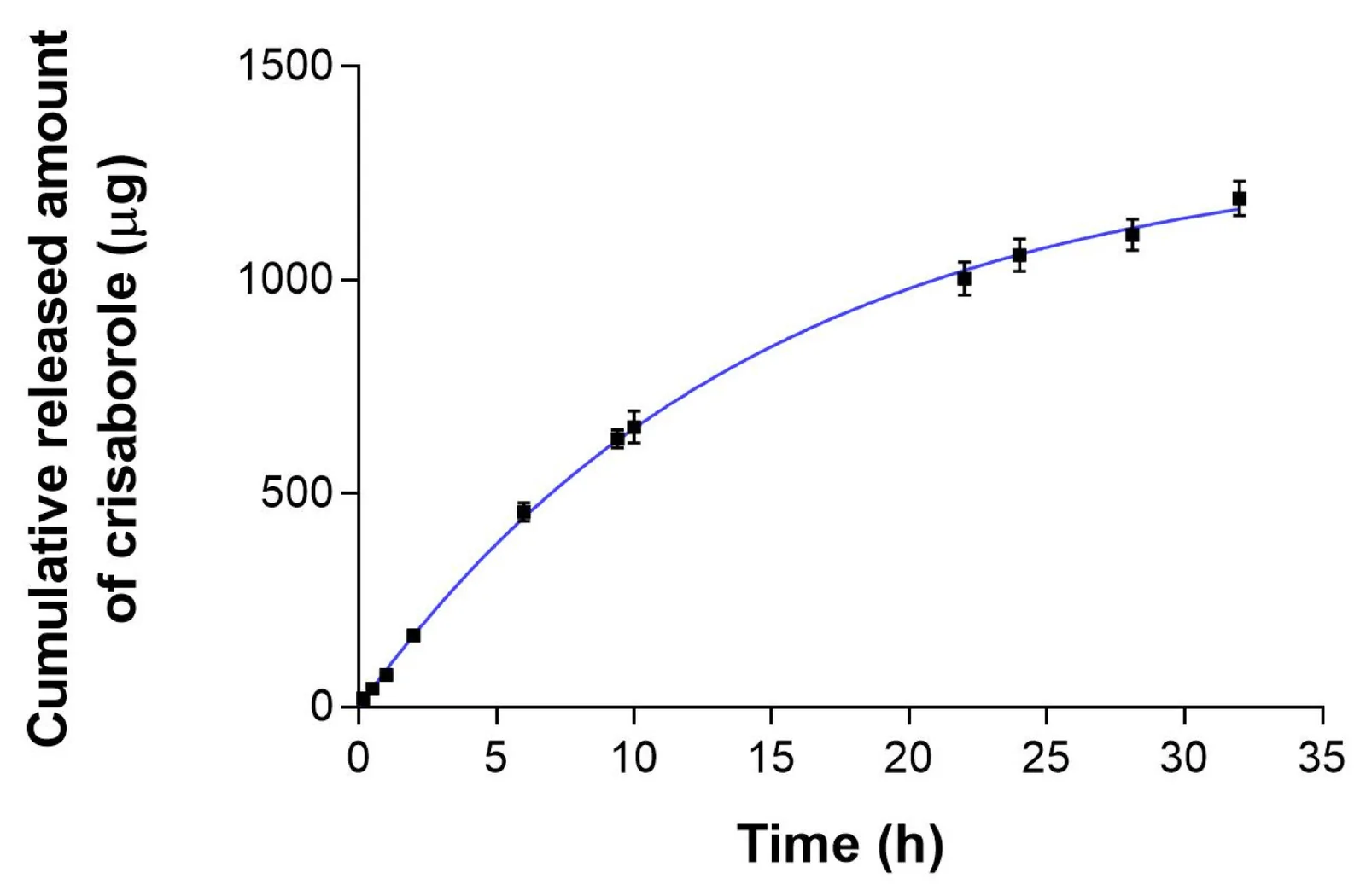
In vitro release profile of CRB from CRB-NE through a dialysis membrane. Data are expressed as mean ± SD (*n* = 6).

As shown in Figure 8, cumulative CRB release reached approximately 1.2 mg at the end of the experiment, corresponding to nearly 80% of the amount initially loaded into the donor compartment. Regression analysis indicated that the release profile was best fitted by a first-order kinetic model (R^2^ = 0.99), with a release rate constant (k) of 0.07 h^−1^ and a half-life of 10.06 h.

### 3.7. Ex Vivo Permeation Study

**Figure 9:**
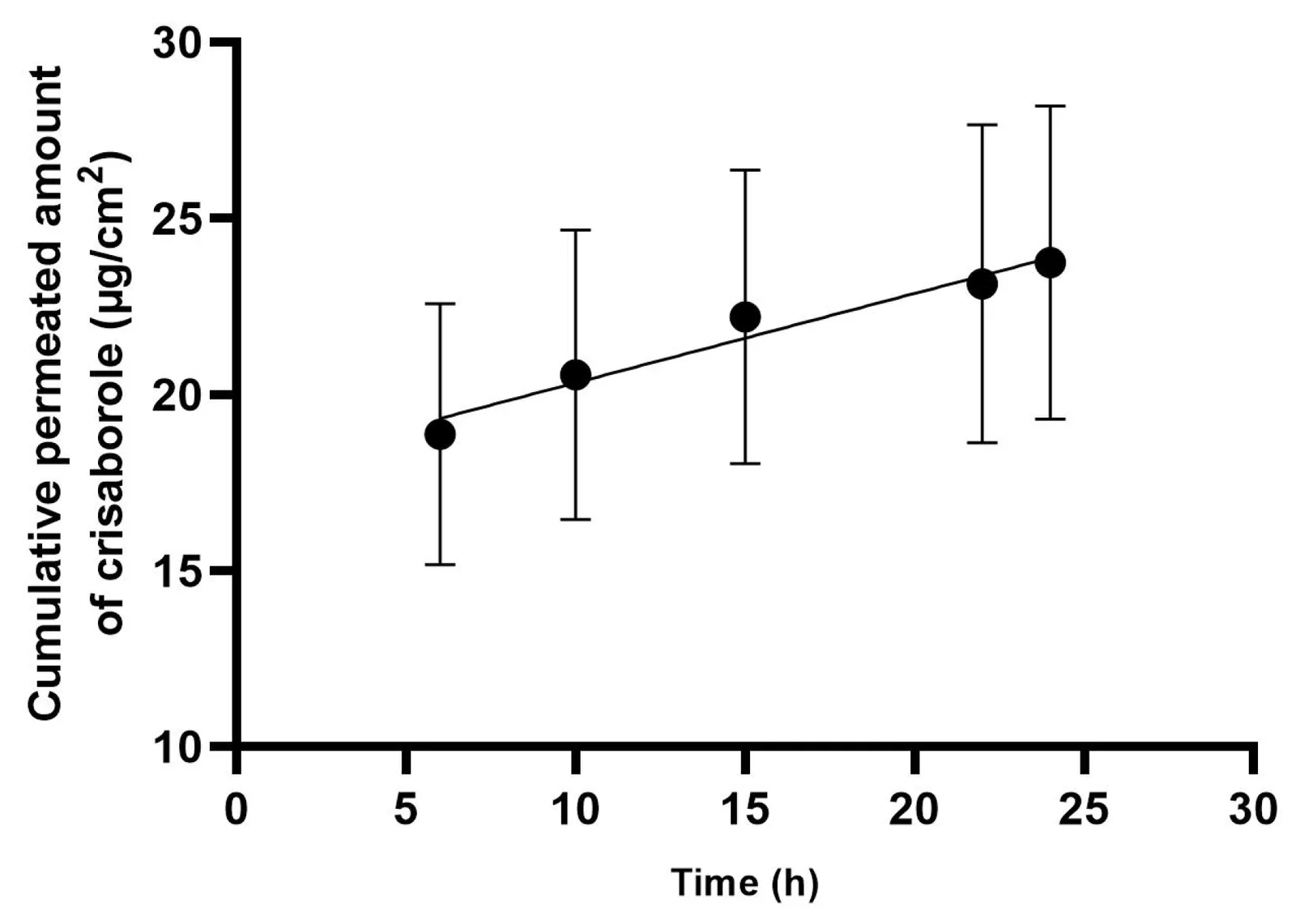
Ex vivo permeation profile of CRB from CRB-NE through human skin. Cumulative amount of CRB permeated per unit area (μg/cm^2^) is plotted as a function of time. Data are expressed as mean ± SD (*n* = 6).

The ex vivo permeation profile of CRB from CRB-NE through human skin is shown in Figure 9. The cumulative amount of CRB permeated progressively increased over time, reaching 23.75 ± 4.44 μg/cm^2^ after 24 h. Analysis of the permeation profile yielded a steady-state flux (*J*_ss_) of 0.25 ± 0.04 μg/cm^2^/h and a permeability coefficient (*K*_p_) of (5.10 ± 0.71) × 10^−5^ cm/h. In parallel, a substantial amount of CRB was retained within the skin after 24 h (*Q*_ret_ = 1098.43 μg/g skin/cm^2^).

### 3.8. Safety Evaluation

#### 3.8.1. In Vitro Cytotoxicity by MTT (3-(4,5-Dimethylthiazol-2-yl)-2,5-Diphenyltetrazolium Bromide) Assay

**Figure 10:**
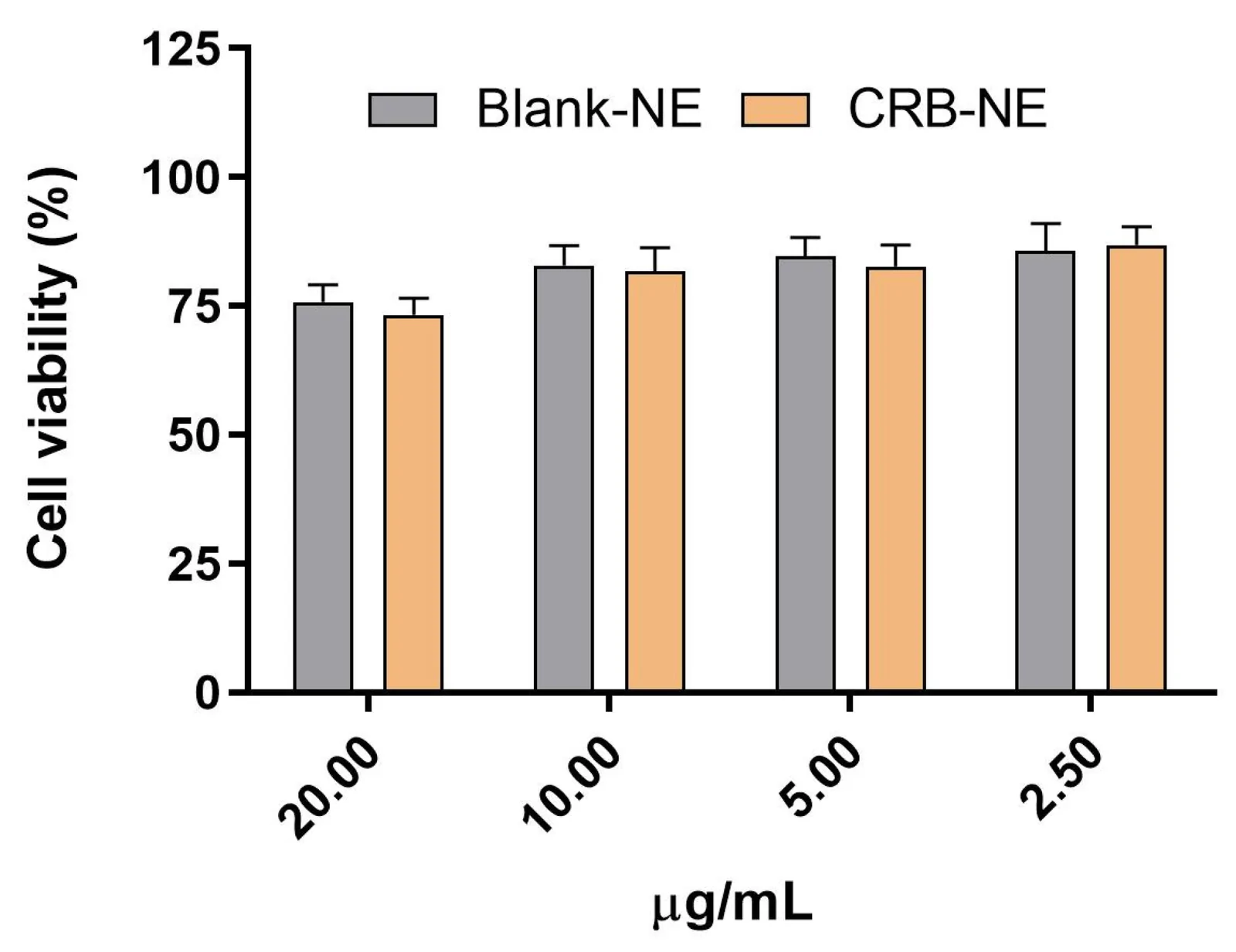
Cell viability of HaCaT keratinocytes after exposure to CRB-NE and Blank-NE, determined by the MTT assay. Data are expressed as mean ± SD (*n* = 3).

Cytotoxicity assays in HaCaT keratinocytes revealed that both CRB-NE and Blank-NE maintained cell viability above 80% at concentrations ranging from 2.5 to 10 µg/mL (Figure 10). Furthermore, no statistically significant differences were detected between the two formulations across the tested concentration range, indicating that neither the nanoemulsion excipients nor CRB incorporation adversely affected keratinocyte viability under the experimental conditions employed.

#### 3.8.2. In Vitro HET-CAM Assay

**Figure 11:**
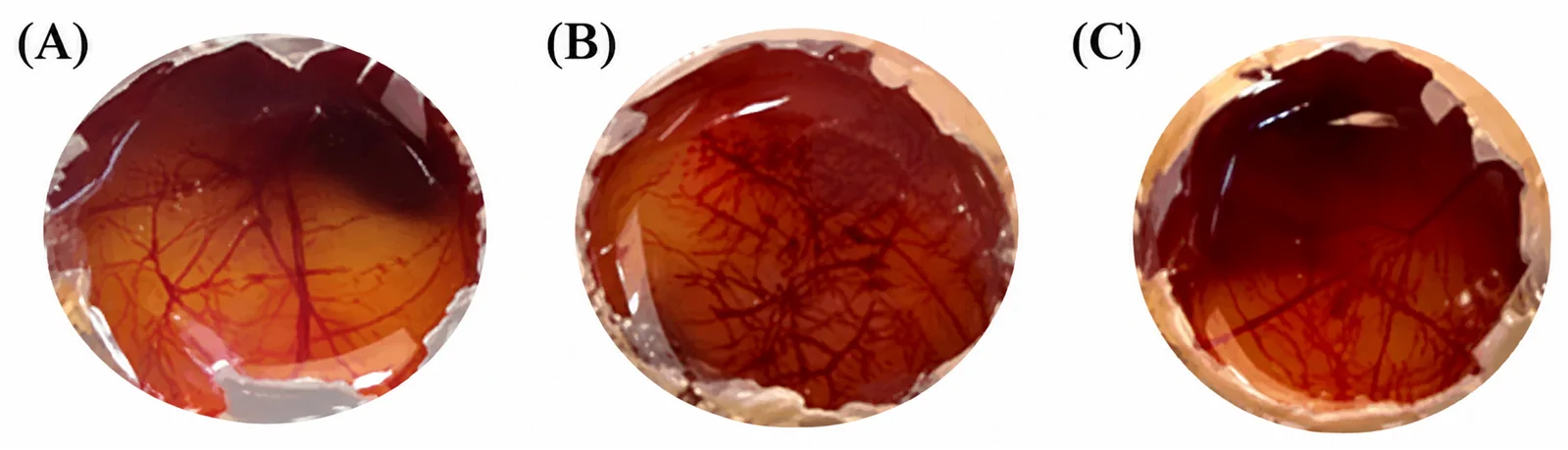
HET-CAM assay for the evaluation of the irritation potential of CRB-NE. Representative images of the chorioallantoic membrane after 5 min of exposure to: (**A**) Negative control (0.9% NaCl), (**B**) Positive control (0.1 N NaOH), and (**C**) CRB-NE.

Figure 11 shows the irritancy potential of CRB-NE evaluated by HET CAM assay. The positive control induced marked vascular responses, including vasodilation and severe hemorrhage, which progressively intensified throughout the 5 min observation period, resulting in its classification as a moderate irritant with an irritation score (IS) of 7.64 ± 0.23. Conversely, CRB-NE produced no detectable signs of irritation on the chorioallantoic membrane and yielded an irritation score (IS) of 0.78 ± 0.35, indicating that the formulation can be considered non-irritant.

#### 3.8.3. Skin Tolerance and Barrier Function Assessment

**Figure 12:**
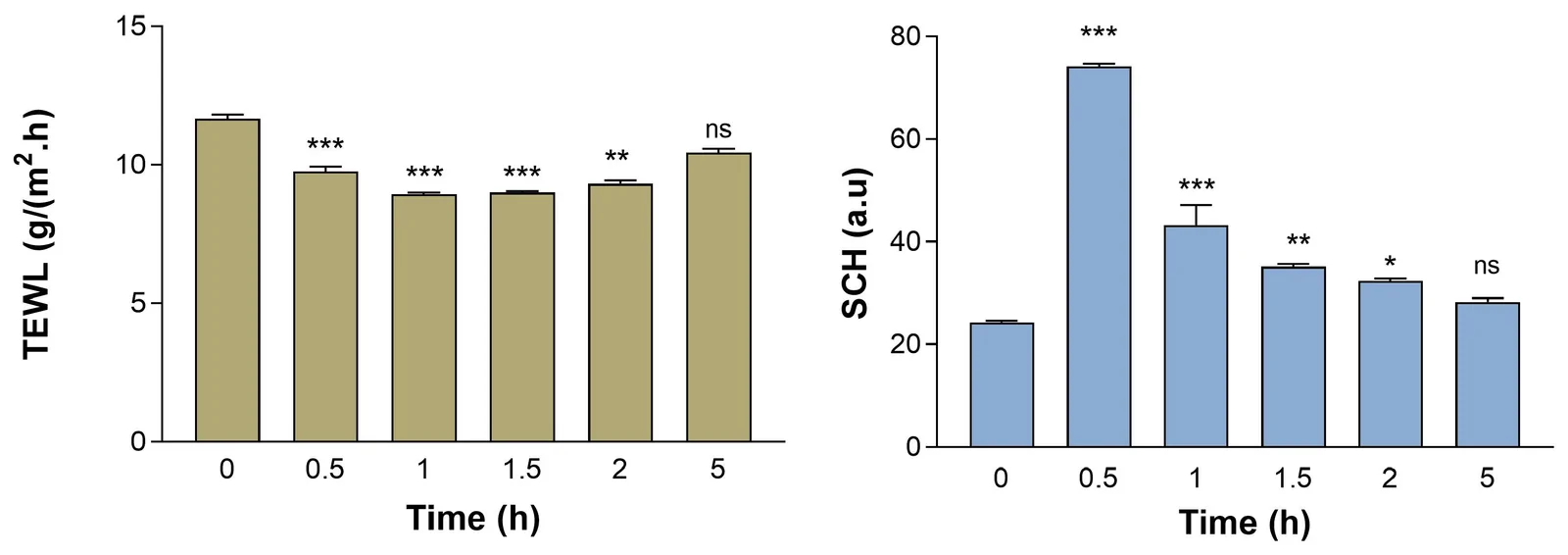
Effect of Blank-NE on skin barrier integrity (TEWL and SCH) in healthy volunteers. Values represent mean ± SD (*n* = 10). Statistically significant differences compared to the baseline state: *, *p* < 0.05; **, *p* < 0.01; ***, *p* < 0.001; ns, not significant.

The evaluation of skin barrier function after topical application of Blank-NE revealed a significant decrease in transepidermal water loss (TEWL) accompanied by a concomitant increase in stratum corneum hydration (SCH) 0.5 h after application (*p* < 0.05). Both parameters progressively returned to baseline levels after 5 h, suggesting that the formulation temporarily enhanced skin hydration without adversely affecting barrier integrity (Figure 12).

### 3.9. Anti-Inflammatory Efficacy

#### 3.9.1. In Vitro Anti-Inflammatory Efficacy

**Figure 13:**
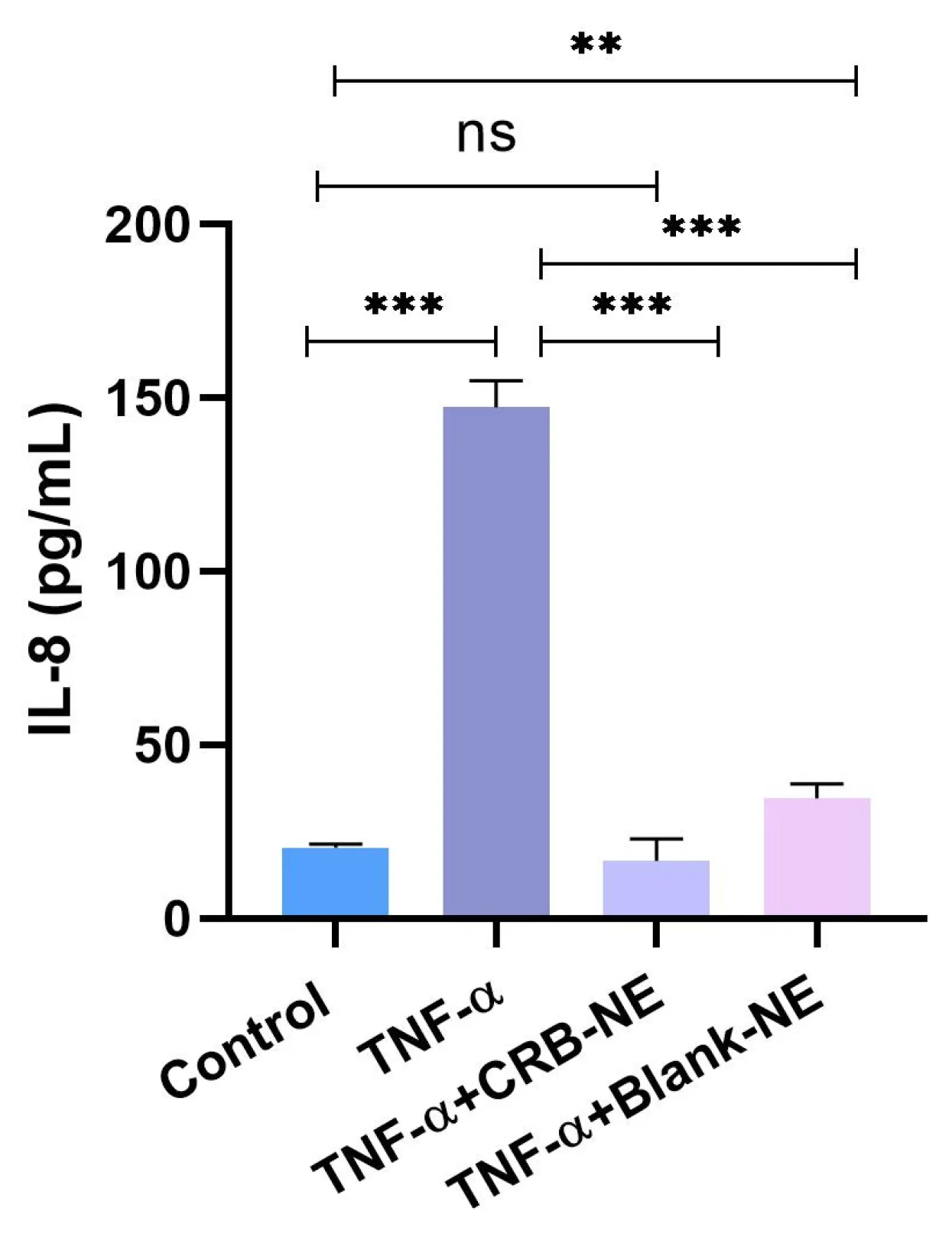
In vitro anti-inflammatory activity of CRB-NE evaluated by IL-8 secretion in HaCaT cells. Data are expressed as mean ± SD of three independent experiments. Statistical significance was determined by one-way ANOVA followed by Tukey’s multiple comparison test. ns, not significant; **, *p* < 0.01; ***, *p* < 0.001.

The anti-inflammatory activity of CRB-NE was assessed by quantifying IL-8 secretion in TNF-α-stimulated HaCaT cells. As shown in Figure 13, TNF-α markedly increased IL-8 production compared with untreated cells. Treatment with CRB-NE (5 μg/mL) significantly reduced IL-8 secretion, demonstrating the capacity of the nanoemulsion to modulate the inflammatory response in activated keratinocytes.

#### 3.9.2. In Vivo Anti-Inflammatory Efficacy

**Figure 14:**
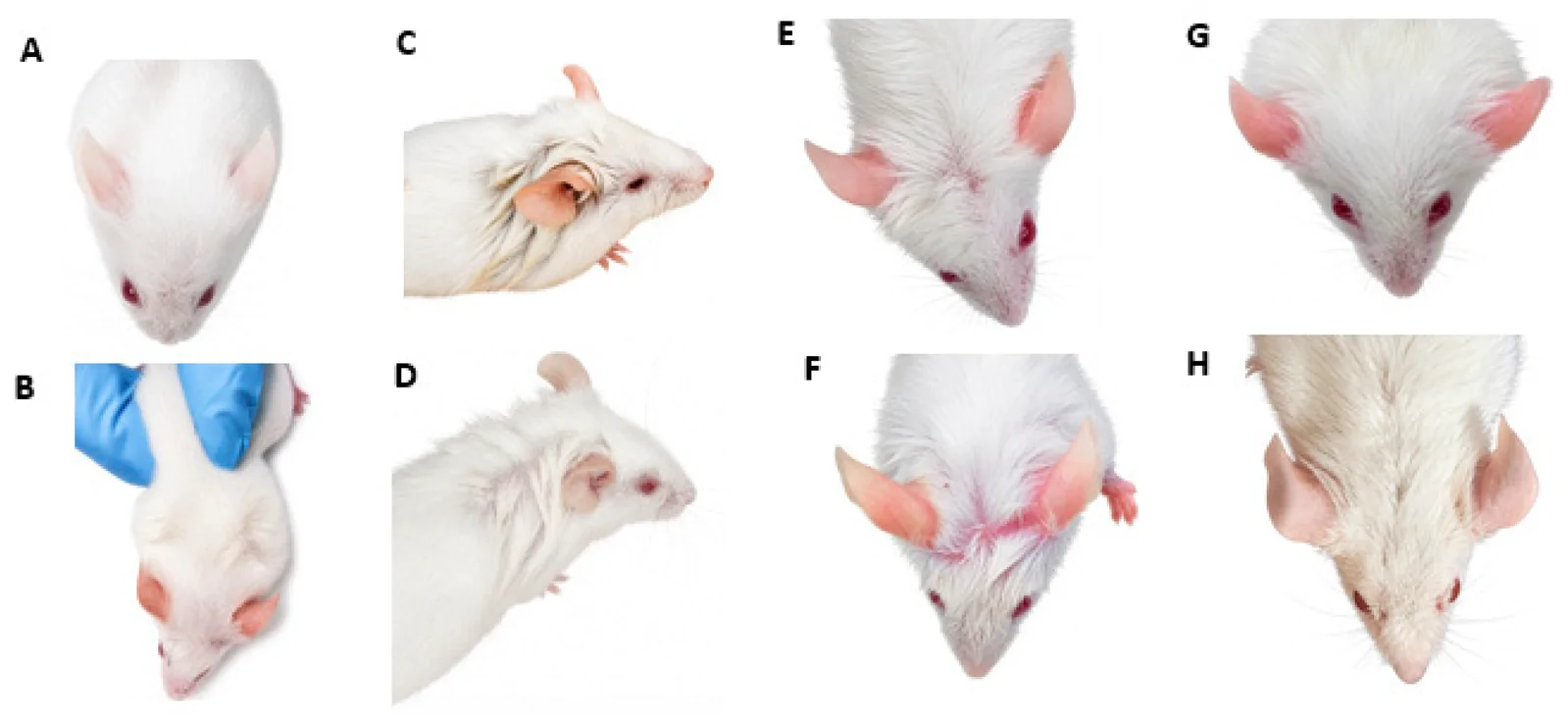
Representative macroscopic images of xylene-induced ear inflammation in mice following topical treatment. (**A**) Healthy untreated mouse (negative control). (**B**) Xylene-treated mouse (inflamed positive control). (**C**,**D**) Ear before and after treatment with CRB-NE, respectively. (**E**,**F**) Ear before and after treatment with Blank-NE, respectively. (**G**,**H**) Ear before and after treatment with commercial diclofenac gel, respectively. Representative images illustrate the reduction in xylene-induced erythema following treatment with CRB-NE and diclofenac gel.

Representative images of the macroscopic evaluation of xylene-induced ear inflammation are shown in Figure 13. The untreated healthy mouse (Figure 14A) exhibited no visible signs of erythema or tissue damage, whereas the xylene-treated control (Figure 14B) showed marked erythema similar to that generated in rosacea, confirming successful induction of acute inflammation. Figure 14C,D illustrate the ear before and after treatment with CRB-NE, respectively, showing a noticeable reduction in xylene-induced erythema. A similar macroscopic improvement was observed following treatment with the commercial diclofenac gel (Figure 14G,H). In contrast, ears treated with Blank-NE (Figure 14E,F) exhibited no evident reduction in erythema or no visible anti-inflammatory effect.

**Figure 15:**
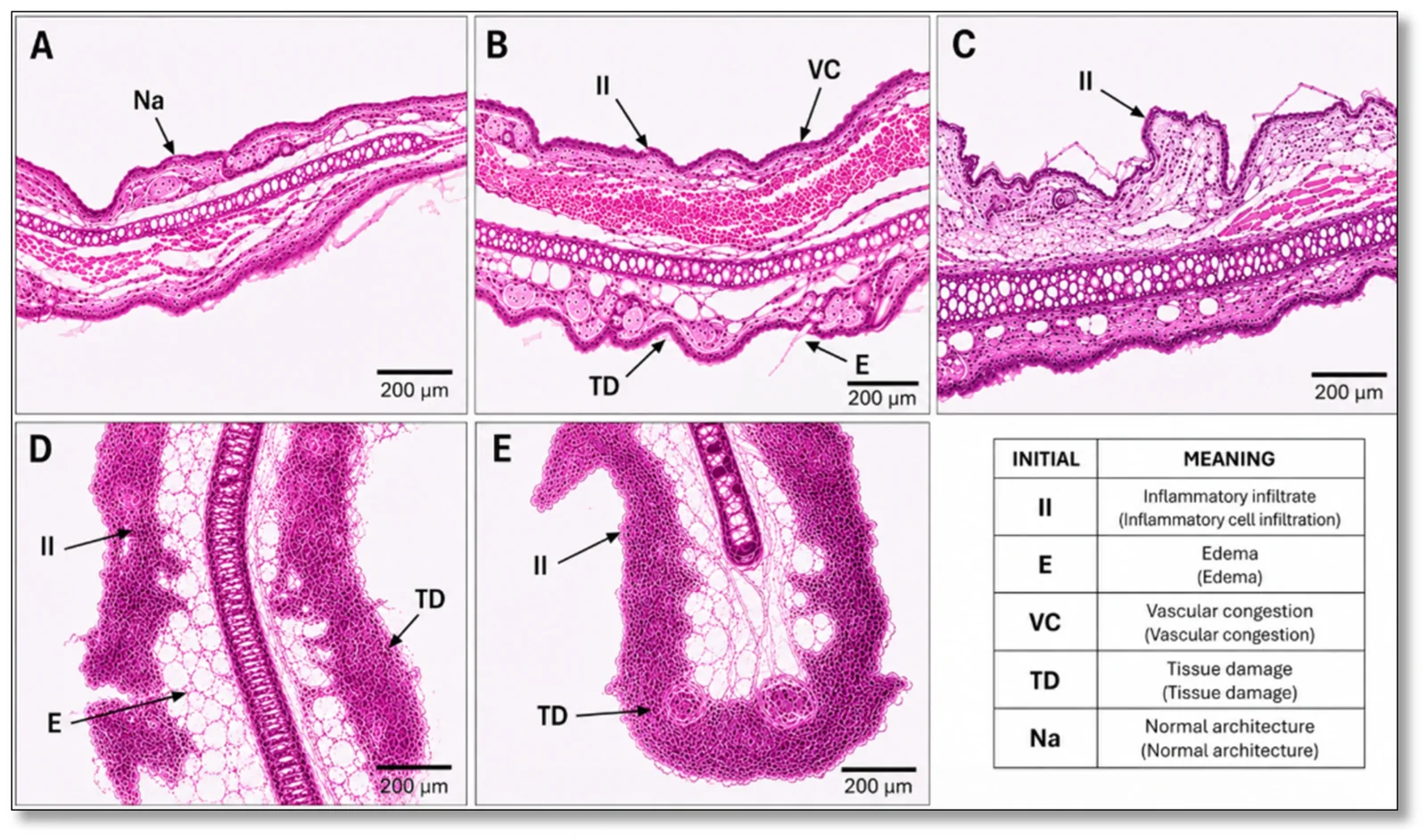
Representative histological images of H&E-stained skin sections (100× magnification). (**A**) Negative control group showing preserved normal architecture. (**B**) Positive control group with inflammatory infiltrate, vascular congestion, edema, and tissue damage. (**C**) Group treated with CRB-NE showed a reduction in inflammatory infiltrate and partial restoration of tissue morphology. (**D**) Group treated with Blank-NE showing persistent inflammatory changes and tissue damage. (**E**) Group treated with commercial diclofenac gel showed moderate improvement in inflammatory lesions. Scale bar = 200 μm. Abbreviations: II, inflammatory infiltrate; E, edema; VC, vascular congestion; TD, tissue damage; Na, normal architecture.

Histopathological examination of hematoxylin and eosin-stained ear sections revealed clear differences among the experimental groups. Inflammatory infiltrate, edema, vascular congestion, and tissue alterations were reported by two blinded evaluators (Table 3). The negative control group (Figure 15A) exhibited preserved tissue architecture, characterized by an intact epidermis, an organized dermis, and the absence of evident inflammatory cell infiltration or edema. In contrast, the xylene-treated control group (Figure 15B) displayed pronounced histopathological alterations, including vascular congestion, extensive inflammatory cell infiltration within the dermis, interstitial edema, and disruption of normal tissue architecture, confirming the establishment of the inflammatory response. Treatment with CRB-NE (Figure 15C) markedly attenuated the histopathological alterations induced by xylene. Reduced vascular congestion and inflammatory cell infiltration, together with decreased edema and improved preservation of tissue architecture, were observed compared with the inflamed control. In contrast, the Blank-NE-treated group (Figure 15D) showed persistent inflammatory cell infiltration, edema, and tissue alterations, indicating that the vehicle alone exerted a limited effect on the xylene-induced inflammatory response. The diclofenac-treated group (Figure 15E) also exhibited an improvement in tissue morphology, with reduced inflammatory infiltration and edema and better preservation of tissue organization compared with the inflamed control, although residual signs of inflammation remained. These results were consistent with the macroscopic observations and support the anti-inflammatory activity of CRB-NE in the xylene-induced ear inflammation model.

## 4. Discussion

In this study, the incorporation of crisaborole (CRB) into a topical nanoemulsion (CRB-NE) was investigated as a formulation strategy to enhance its dermal delivery for the treatment of atopic dermatitis. Selection of the nanoemulsion components was guided by their ability to solubilize CRB and their suitability for topical administration. Lauroglycol^®^ 90, Tween^®^ 80, and Transcutol^®^ P exhibited the highest solubilizing capacity among the oils, surfactants, and co-surfactants evaluated, respectively. In particular, Lauroglycol^®^ 90 is a water-insoluble, lipophilic excipient which consists of monoesters and a small fraction of diesters of lauric acid, Tween^®^ 80 is as a nonionic oil-in-water (O/W) surfactant with a hydrophilic–lipophilic balance (HLB) of 15, and Transcutol^®^ P is a hydrophilic solvent widely used in dermal formulations because of its high solubilizing capacity and its ability to facilitate drug partitioning into the skin [23]. The resulting formulation (CRB-NE), containing 0.5% CRB, exhibited a pH of approximately 5.0, close to the naturally acidic environment of the skin and therefore suitable for topical application [24]. Physicochemical characterization confirmed the formation of nanosized droplets, with a mean diameter of 105.3 nm and a relatively narrow size distribution (PDI = 0.287), while TEM analysis revealed predominantly spherical droplets (Figure 3). The small droplet size provides a large interfacial surface area and close contact with the skin surface, properties that may favor CRB release and partitioning into the stratum corneum [25].

For topical drug delivery, rheological and spreading properties are important determinants of formulation performance, as they influence handling, dosing, application, and distribution over the skin surface and may also affect drug release from the vehicle [26]. CRB-NE exhibited a linear relationship between shear stress and shear rate, while viscosity remained essentially constant over the investigated shear rate range (Figure 4). This behavior was further supported by mathematical modeling, which showed an excellent fit to the Newtonian model. The relatively low viscosity of CRB-NE (38.32 ± 0.04 mPa·s) is consistent with the fluid nature typically observed for nanoemulsion systems and may facilitate uniform distribution of the formulation over the skin surface. Consistent with its low viscosity, CRB-NE exhibited good spreadability, with the spreading area progressively increasing as a function of the applied load and reaching approximately 22 cm^2^ (Figure 5). This behavior indicates that relatively low mechanical force is sufficient to distribute the formulation over a large surface area, an advantageous property for topical administration, particularly when application to inflamed or sensitive skin is required [27]. Taken together, the Newtonian flow behavior, low viscosity, and high spreadability of CRB-NE support its suitability as an easily applicable topical formulation and suggest its potential for administration through low-viscosity delivery systems, including spray-based applications [28].

Physical stability is a critical attribute of nanoemulsion systems, since changes in droplet organization during storage may lead to destabilization phenomena such as creaming, sedimentation, flocculation, or coalescence [29,30]. In the present study, multiple light scattering analysis revealed no relevant changes in the transmission profiles of CRB-NE during 60 days of storage at 4, 25, and 40 °C (Figure 6), indicating that the nanoemulsion maintained good physical stability under all tested conditions. This behavior may be partly attributed to the small droplet size of the system, which favors Brownian motion and reduces the tendency of dispersed droplets to undergo gravitational separation [31]. In addition, Tween^®^ 80, as a nonionic surfactant, may contribute to stabilization by providing a steric barrier around the droplets, thereby reducing droplet aggregation and coalescence [23,32]. Regarding chemical stability, CRB-NE maintained relatively stable pH values and CRB content during storage at 25 °C for 90 days, suggesting adequate compatibility between the drug and the formulation components under these conditions (Figure 7). In contrast, significant changes in both parameters were observed during storage at 40 °C, particularly after two months, indicating that elevated temperature may accelerate physicochemical changes or CRB degradation within the formulation. Taken together, these findings demonstrate that although CRB-NE remained physically stable across the investigated temperature range, its chemical stability was more sensitive to prolonged exposure to elevated temperature. These results support storage under controlled temperature conditions and highlight the need for longer-term stability studies to establish the optimal storage conditions and shelf life of the formulation.

In vitro release studies provide valuable information on the performance of drug delivery systems and may serve as a useful tool for formulation characterization and quality control. Drug release from a topical formulation is governed by the physicochemical properties of the drug and its interactions with the vehicle, and represents a prerequisite for its subsequent partitioning into and permeation through the skin [33]. In the present study, CRB-NE exhibited a progressive release profile, reaching approximately 1.2 mg of CRB, corresponding to nearly 80% of the initially loaded drug, by the end of the experiment (Figure 8). Among the kinetic models evaluated, the experimental data were best described by a first-order model (R^2^ = 0.99), with a release rate constant (k) of 0.07 h^−1^ and a half-life (t_1/2_) of 10.06 h. First-order kinetics indicates that the release rate is dependent on the amount of drug remaining within the formulation, resulting in a progressively decreasing release rate as CRB is depleted from the nanoemulsion [34]. Overall, the high fraction of CRB released demonstrates that the drug is readily available for release from CRB-NE, an essential step preceding its partitioning and subsequent penetration into the skin.

Ex vivo permeation studies using human skin revealed a marked preferential localization of CRB within the skin. After 24 h, only 23.71 μg of CRB reached the receptor compartment, whereas a substantially higher amount was retained within the skin (*Q*_ret_ = 1098.43 μg/g skin/cm^2^). This permeation–retention profile is particularly relevant to achieve localized anti-inflammatory therapy for the topical treatment of atopic dermatitis [35]. Thus, the high cutaneous retention observed for CRB-NE suggests that the formulation may provide a local drug reservoir while limiting the fraction of CRB that crosses the entire skin membrane and reaches the receptor compartment. The preferential skin accumulation of CRB may be related, at least in part, to the composition and nanometric characteristics of the formulation. In particular, Transcutol^®^ P is widely recognized as a solvent and skin penetration enhancer capable of partitioning into the stratum corneum and modifying the local environment of its lipid and protein domains. Moreover, its interaction with the stratum corneum can increase drug solubility and partitioning within the skin [36]. Thus, the combination of efficient drug solubilization, the large interfacial area provided by the nanosized droplets, and the presence of Transcutol^®^ P may therefore contribute to the high skin retention observed while maintaining limited passage into the receptor compartment [37]. Although the ex vivo permeation results indicate preferential cutaneous retention of CRB, the absence of in vivo pharmacokinetic and biodistribution studies prevents conclusions regarding systemic exposure, tissue distribution, and drug elimination. Further investigations are required to establish the in vivo disposition of CRB following topical administration of CRB-NE. A further limitation is the absence of a direct ex vivo comparison with a marketed 2% crisaborole ointment, which was not available for this study. Consequently, the present findings do not establish whether CRB-NE provides superior cutaneous delivery or retention compared with the conventional formulation. Such comparisons should be addressed in future studies.

The safety and tolerability of topical formulations are particularly relevant for the treatment of atopic dermatitis, where the skin barrier is already compromised and patients may be more susceptible to irritation [38]. Cytotoxicity assessment in HaCaT keratinocytes showed that both CRB-NE and Blank-NE maintained cell viability above 80% at concentrations ranging from 2.5 to 10 μg/mL after 24 h of exposure (Figure 10). Moreover, no significant differences in cell viability were observed between CRB-NE and Blank-NE, suggesting that neither the nanoemulsion excipients nor CRB incorporation adversely affected keratinocyte viability under the experimental conditions evaluated. The absence of a free CRB control limits direct assessment of whether nanoencapsulation modifies the intrinsic cytotoxicity of the drug. Nevertheless, comparison with Blank-NE allowed evaluation of the contribution of the formulation vehicle to cell viability. The low irritation potential of the formulation was further supported by the HET-CAM assay (Figure 11). This model provides a sensitive approach for evaluating the local irritation potential of formulations through direct observation of vascular responses in the highly vascularized chorioallantoic membrane, including hemorrhage, vascular lysis, and coagulation [39]. In the present study, CRB-NE produced no relevant vascular alterations and yielded an irritation score of 0.78, corresponding to a non-irritant classification. This finding is particularly relevant considering the presence of surfactant and co-surfactant in the nanoemulsion, since these components may potentially affect biological membranes depending on their nature and concentration [40]. The absence of a relevant response in the HET-CAM model therefore suggests that the combination and proportions of excipients used in CRB-NE were well tolerated under the experimental conditions. The effect of the nanoemulsion vehicle on human skin barrier function was further investigated by monitoring transepidermal water loss (TEWL) and stratum corneum hydration (SCH) following topical application of Blank-NE in healthy volunteers. TEWL reflects water diffusion through the epidermis and is widely used as a non-invasive indicator of skin barrier integrity, whereas SCH provides information on the hydration state of the stratum corneum. Disruption of the cutaneous barrier is generally associated with increased TEWL and may be accompanied by alterations in stratum corneum hydration [41]. In the present study, Blank-NE did not adversely affect these biophysical parameters. Instead, a transient significant decrease in TEWL accompanied by an increase in SCH was observed shortly after application, with both parameters progressively returning toward baseline values (Figure 12). This response suggests that the nanoemulsion vehicle did not compromise skin barrier integrity and may exert a transient moisturizing or occlusive effect. Taken together, the preservation of keratinocyte viability, the low irritation score in the HET-CAM assay, and the absence of detrimental changes in human skin barrier parameters provide complementary evidence supporting the favorable tolerability profile of the CRB-NE system. These findings are particularly relevant for its intended topical application in atopic dermatitis, although further studies in diseased skin and under repeated-use conditions would be required to establish its long-term cutaneous tolerability.

The xylene-induced ear inflammation model successfully generated an acute inflammatory response, as demonstrated by the marked macroscopic and histopathological alterations observed in the inflamed control group. Xylene induces a rapid local inflammatory response characterized by vasodilation, increased vascular permeability, edema, erythema, and inflammatory cell recruitment [42]. Accordingly, xylene-treated animals exhibited pronounced erythema and histopathological alterations, including vascular congestion, interstitial edema, inflammatory cell infiltration, and disruption of normal tissue architecture compared with healthy controls. Topical treatment with CRB-NE markedly attenuated these inflammatory changes. Macroscopically, a reduction in xylene-induced erythema was observed following treatment (Figure 14), while histopathological examination revealed decreased vascular congestion, edema, and inflammatory cell infiltration, together with improved preservation of tissue architecture (Figure 15). These findings are consistent with the known pharmacological activity of CRB as a phosphodiesterase-4 (PDE4) inhibitor [43]. The in vivo findings were also consistent with the anti-inflammatory activity observed in TNF-α-stimulated HaCaT keratinocytes. CRB-NE significantly reduced IL-8 secretion in this cellular model (Figure 13), providing complementary evidence that the formulation can modulate inflammatory mediator production. IL-8 is a potent chemokine involved in neutrophil recruitment and activation and contributes to the amplification of cutaneous inflammatory responses [44,45]. Therefore, the concordance between reduced IL-8 production in vitro and attenuation of inflammatory alterations in vivo supports the anti-inflammatory activity of CRB delivered through the nanoemulsion. Importantly, Blank-NE produced little apparent improvement in the macroscopic and histopathological features of xylene-induced inflammation compared with CRB-NE. This observation suggests that the therapeutic response was primarily associated with the pharmacological activity of CRB rather than with an intrinsic anti-inflammatory effect of the nanoemulsion vehicle. Diclofenac gel, included as a reference topical anti-inflammatory treatment, also reduced the inflammatory alterations induced by xylene. CRB-NE showed a histological improvement of a magnitude comparable to that observed with diclofenac. The lack of a free CRB comparator in the anti-inflammatory assays prevents direct determination of whether nanoemulsification enhances the pharmacological efficacy of CRB. However, the inclusion of Blank-NE as a vehicle control supports the attribution of the observed anti-inflammatory effects to the CRB-loaded formulation rather than the vehicle alone. Taken together, the in vitro and in vivo findings provide complementary evidence that CRB-NE effectively attenuates inflammatory responses. Nevertheless, given the acute and nonspecific nature of the xylene-induced inflammation model, further studies using experimental models that more closely reproduce the chronic barrier dysfunction and immune alterations characteristic of atopic dermatitis would be valuable to confirm the therapeutic potential of CRB-NE for this indication.

## 5. Conclusions

This study demonstrates the feasibility of a crisaborole-loaded nanoemulsion (CRB-NE) as a dermal delivery system for the topical treatment of atopic dermatitis. The optimized formulation exhibited suitable physicochemical and rheological properties, good spreadability, and adequate physical stability, while enabling efficient release of CRB from the vehicle. Importantly, ex vivo studies in human skin revealed preferential cutaneous localization of CRB, with substantial drug retention and limited passage across the entire skin membrane, a delivery profile particularly advantageous for a drug intended to exert its pharmacological action locally. The formulation also showed a favorable tolerability profile across complementary cellular, HET-CAM, and human skin barrier assessments. Beyond its pharmaceutical performance, CRB-NE demonstrated biological activity by reducing IL-8 secretion in activated keratinocytes and attenuating macroscopic and histopathological alterations in an in vivo model of acute cutaneous inflammation. Collectively, these findings establish a coherent relationship between drug release, cutaneous retention, tolerability, and anti-inflammatory activity, supporting CRB-NE as a promising strategy for localized delivery of CRB. Further studies in disease-specific models of atopic dermatitis and under repeated-dose conditions are warranted to confirm its therapeutic performance and support its future development as a topical treatment.

## Figures and Tables

**Table 1:** Interpretation of HET-CAM test results.

Irritation Index	Irritation Categories
0–0.9	No irritation
1–4.9	Mild irritation
5–8.9	Moderate irritation
9–21	Severe irritation

**Table 2:** Final composition of CRB-NE.

Components	%
Crisaborole	0.5
Lauroglycol^®^ 90	6
Tween^®^ 80	12.25
Transcutol^®^	36.75
Water	44.5

**Table 3:** Semi-quantitative histopathological scoring of skin inflammation in the different experimental groups.

Parameter	A	B	C	D	E
Inflammatory infiltrate	0.00 + 0.00	3.00 + 0.00	0.75 + 0.25	3.00 + 0.00	2.00 + 0.00
Edema	0.00 + 0.00	3.00 + 0.00	0.75 + 0.25	2.00 + 0.25	0.75 + 0.25
Vascular congestion	0.00+ 0.00	3.00 + 0.00	1.00 + 0.00	2.00 + 0.25	1.00 + 0.25
Tissue damage	0.25 + 0.25	3.00 + 0.00	0.75 + 0.25	3.00 + 0.00	2.00 + 0.00
Normal architecture	2.50 + 0.50	0.00 + 0.00	2.00 + 0.00	0.00 + 0.00	2.00 + 0.25

Scale: 0 = absent, 1 = mild, 2 = moderate, 3 = severe.

## Data Availability

The data presented in this study are available in this article.

## Supplementary Materials

The following supporting information can be downloaded at: https://www.mdpi.com/article/10.3390/pharmaceutics18101342/s1, Figure S1: Linearity of the analytical method for CRB. The result is expressed as the mean and standard deviation of n = 6 calibration curves; Figure S2: Specificity of the analytical method for CRB. (A) CRB extracted from human skin after the permeation study; (B) 50 μg/mL standard; (C) blank skin as a control and (D) blank mobile phase as a control; Table S1: Standard CRB curve and respective area response factor; Table S2: Accuracy and precision of the analytical method for CRB solutions; Table S3: Robustness of the analytical method by evaluating variations in mobile phase composition; Table S4: Sensitivity of analytical method for identification and quantification of CRB. Table S5: Composition of the six CRB-loaded candidate nanoemulsions evaluated during formulation optimization.
