# Supplementary material for: Crisaborole-Loaded Nanoemulsion for Atopic Dermatitis: Localized Dermal Delivery of a Phosphodiesterase-4 Inhibitor
Source: Pharmaceutics. 2026 Oct 21;18(10):1342. doi: 10.3390/pharmaceutics18101342 (PMC13708513; doi:10.3390/pharmaceutics18101342)
Supplement: Supplementary file 1 [file pharmaceutics-18-01342-s001.zip › pharmaceutics-4591122-supplementary.pdf]

Supplementary Materials

Validation of the Analytical Method

Linearity was evaluated from six calibration curves within a range of 1 to 100 μg/mL (Table S1). No points were left out of the calibration during validation and the data showed good linearity of the proposed method (Figure S1). The r^2 value in each of the calibration curves was >0.9999, indicating a linear relationship between analyte concentration and peak area. No statistically significant differences were found after one-way analysis of variance test (one-way ANOVA) of the calibration curves, where the *p value = 0.583*.

Table S1. Standard CRB curve and respective area response factor.

Standard concentration (μg/ml)	Ratio 1	Ratio 2	Ratio 3	Ratio 4	Ratio 5	Ratio 6
1	7.554	4.391	4.044	4.119	3.839	4.125
5	5.250	4.290	4.659	4.558	4.090	5.561
10	5.243	4.127	4.564	4.500	3.944	5.507
25	5.156	4.030	4.594	4.475	4.121	5.416
50	5.126	4.265	4.493	4.442	4.067	5.375
100	5.062	4.253	4.419	4.318	3.959	5.279

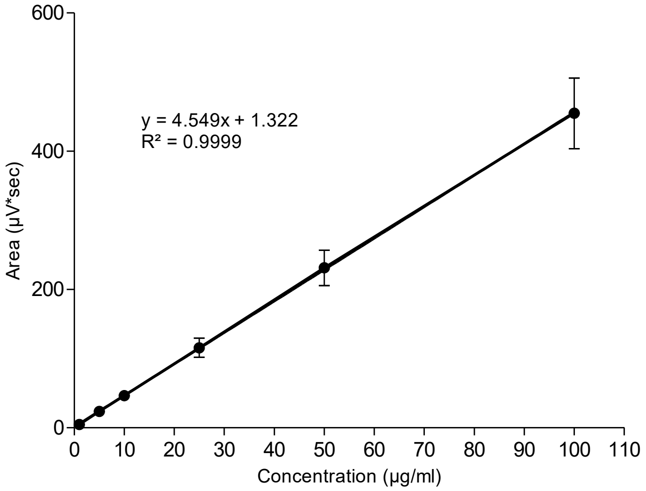

Figure S1. Linearity of the analytical method for CRB. The result is expressed as the mean and standard deviation of n=6 calibration curves.

Precision and accuracy values were obtained from a standard APR concentration ranging from 1 to 100 μg/mL. Inter-day precision and accuracy were calculated after analyzing samples on three different days. The results were presented in Tables S2. These results showed good precision with RSD values below 4.35%, while the accuracy showed a maximum RE of 1.69%.

Table S2. Accuracy and precision of the analytical method for CRB solutions

Standard Concentration (μg/ml)	Calculated Concentration (μg/ml)	RSD%	RE%

1.0	0.98 ± 0.04	4.13	1.67
5.0	4.92 ± 0.21	4.35	1.69
10.0	9.91 ± 0.17	1.70	0.87
25.0	25.16 ± 0.63	2.50	-0.65
50.0	50.58 ± 0.27	0.53	-1.16
100.0	99.68 ± 0.24	0.24	0.32

Measurement of robustness allows recognition of the effect of operating parameters on the results and provides an indication of applicability during the study. This parameter was determined by evaluating the retention time of the active with the mobile phase concentration change variations shown in Table S3 (n=6). The RE% of variation 2 of the composition involving an acetonitrile:water mixture (69:31 v/v) was 2.44%.

Table S3. Robustness of the analytical method by evaluating variations in mobile phase composition

**Mobile phase**	**Normal Composition**	**Variation 1**	**RE%**	**Variation 2**	**RE%**
**Acetonitrile:Water**	**70:30**	**71:29**		**69:31**	
RT	4.1	4.1	0.00	4.2	2.44

Specificity (Figure S2) was determined by analyzing the CRB extracted from human skin after the permeation study, 50 µg/mL standard, blank skin as a control, and blank mobile phase as a control. No interference was observed in the chromatogram with respect to CRB retention time.

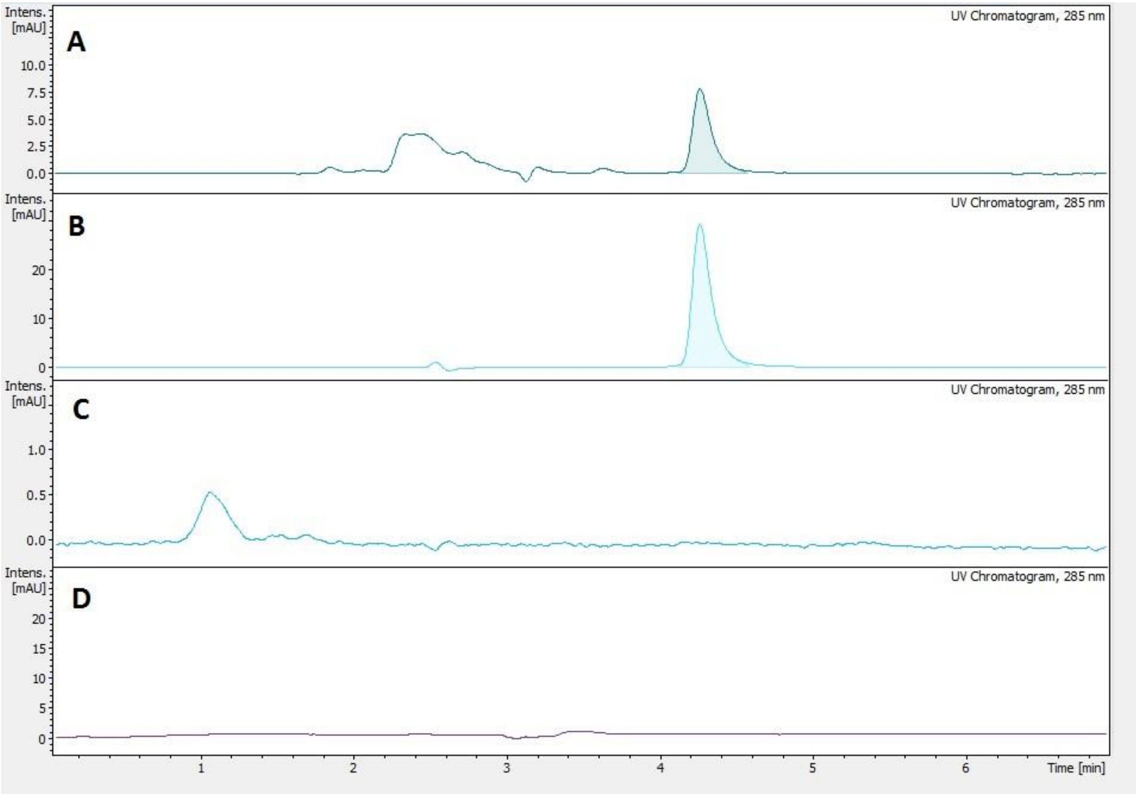

Figure S2. Specificity of the analytical method for CRB. (A) CRB extracted from human skin after the permeation study; (B) 50 µg/mL standard; (C) blank skin as a control and (D) blank mobile phase as a control.

The LOD and LOQ were calculated using the standard deviation of the response and the slope of the calibration curve from 1 to 100 µg/mL, described above. From the flux and the Y-intercept of the six straight lines (Table S4), the LOD for CRB was set at 0.89 ± 0.44 µg/mL and the LOQ at 2.68 ± 1.34 µg/mL. These results indicate that the method is sufficiently sensitive for the determination of CRB.

Table S4. Sensitivity of analytical method for identification and quantification of CRB

n=6	**Mean ± SD**
**LOD**	0.89 ± 0.44
**LOQ**	2.68 ± 1.34

Table S5. Composition of the six CRB-loaded candidate nanoemulsions evaluated during formulation optimization.

**Component (%)**	**F1**	**F2**	**F3**	**F4**	**F5**	**F6**
CRB	0.5	0.5	0.5	0.5	0.5	0.5
Lauroglycol® 90	6	6	6	8	8	8
S_{mix} (Tween® 80:Transcutol® P, 1:3)	49	52	54	52	54	56
Purified water	44.5	41.5	39.5	39.5	37.5	35.5
Total	100	100	100	100	100	100
